# Phosphoprotein enriched in astrocytes (PEA)-15 is a novel regulator of adipose tissue expansion

**DOI:** 10.1038/s41598-021-86250-x

**Published:** 2021-03-26

**Authors:** Pola J. Verschoor, Fiona H. Greig, Justin J. Rochford, Giovanni Levate, Mirela Delibegovic, Dawn Thompson, Alasdair Leeson-Payne, Ruta Dekeryte, Ruth Banks, Joe W. Ramos, Graeme F. Nixon

**Affiliations:** 1 Aberdeen Cardiovascular and Diabetes Centre, Institute of Medical Sciences, University of Aberdeen, Foresterhill, Aberdeen, AB25 2ZD UK; 2 Rowett Institute, University of Aberdeen, Aberdeen, UK; 3 University of Hawaii Cancer Center, University of Hawaii at Manoa, Honolulu, USA

**Keywords:** Biochemistry, Cell biology, Diseases

## Abstract

Excessive expansion of adipose tissue in obesity typically leads to overflow and accumulation of lipids in other tissues, causing fatty liver disease and atherosclerosis. The intracellular protein, phosphoprotein enriched in astrocytes (PEA)-15 has been linked to metabolic disease but its role in lipid storage has not been examined. To delineate the role of PEA-15 in adipose tissue, we placed PEA-15^−/−^ mice on a high fat diet. These mice developed increased body weight and greater white adipose tissue expansion compared to high fat diet-fed wild type mice. This was due to increased adipocyte cell size in PEA-15^−/−^ mice consistent with greater lipid storage capacity. Surprisingly, PEA-15^−/−^ mice exhibited improvements in whole body insulin sensitivity, lower hepatic weight and decreased serum triglycerides indicating a protective phenotype. To determine effects on atherosclerosis, PEA-15^−/−^ mice were crossed with the ApoE^−/−^ mice on a high fat diet. Strikingly, these mice were protected from atherosclerosis and had less hepatic lipid accumulation despite increased adiposity. Therefore, we reveal for the first time that PEA-15 plays a novel role in regulating the expansion of adipose tissue. Decreasing PEA-15 expression increases the sequestering of lipids in adipose tissue, protecting other tissues in obesity, thereby improving metabolic health.

## Introduction

Adipose tissue provides an efficient lipid storage depot, acting as a critical safe energy store and effectively regulating circulating lipid levels by modulating uptake and release^1,2^. Lipid storage mainly occurs in white adipose tissue (WAT), which is principally comprised of unilocular adipocytes specialised for the storage of lipid. However, adipose tissue also contains a wide array of vascular and stromal cells including stem cells from which new adipocytes arise, embedded within a highly regulated extracellular matrix. The properties of adipose tissue and adipocytes vary depending on their location in the body and each depot comprises a mosaic of adipocytes with different stem cell origins and characteristics^1^. This complex tissue also acts as an endocrine organ, secreting several adipokines influencing physiological processes such as insulin sensitivity, appetite and inflammation^1,2^. In obese individuals, persistent nutrient excess can lead to excessive lipid accumulation in adipose depots causing their storage capacity to be overwhelmed^2–6^. This results in overflow of lipid into other metabolically important tissues, particularly the liver^7,8^. This leads to hepatic steatosis which can cause dysregulation of hepatic glucose production and its control by insulin, disrupted cholesterol metabolism and increased circulating plasma lipids, particularly low density lipoprotein^8^. Infiltration and deposition of low density lipoproteins in the arterial wall is the initiating event that ultimately leads to formation of lipid plaques and the development of atherosclerosis^9^. Consequently, obesity and the resultant lipid overflow is now recognised as one of the important factors driving the pathogenesis of lipid-dependent cardiovascular diseases^10,11^.

Delineating the cellular and molecular events leading to lipid overflow from dysfunctional adipose tissue is crucial to understanding the development of many metabolic diseases^2–5^. The expandability of adipose tissue depends on new adipocyte differentiation (hyperplasia) and the ability of the existing adipocytes to further enlarge (hypertrophy)^2–5^. Adipocyte differentiation involves a well-characterised complex transcriptional program initiated by circulating endocrine factors, including adipokines^2,12,13^. Despite much progress in the understanding of adipocyte biology, the regulation of adipose tissue storage efficiency and precisely how the expansion limit of an individual adipocyte is governed remains largely unknown.

Phosphoprotein enriched in astrocytes (PEA)-15, initially cloned from astrocytes, is a cytoplasmic protein that regulates important cellular processes such as proliferation and apoptosis^14^. It is found in many cell types and, via regulation of gene expression, has a role as both an anti-apoptotic and proliferative mediator^14–16^. PEA-15-dependent cellular effects are the result of interactions with PEA-15 binding partners, predominantly the mitogen-activated protein kinase family members, extracellular signal-regulated kinase 1 and 2 (ERK1/2)^14,17^. As PEA-15 can influence important functional cellular outcomes of apoptosis and proliferation, it is not surprising that it has been implicated in several different disease states, notably cancer, cardiovascular disease and diabetes^16,18,19^. In relation to metabolic disease, PEA-15 expression is increased in fibroblasts from type 2 diabetes patients, but not in type 1 diabetes^16^. Despite this link to metabolic dysfunction, the potential role of PEA-15 in adipose tissue and obesity has not been examined in detail, although PEA-15 expression is reportedly increased in adipocytes from mice on a high fat diet^20^.

We hypothesized that PEA-15 could be a physiological regulator of adipocyte development and function. We therefore examined PEA-15^−/−^ mice on a high fat diet. Although this mouse model had increased adiposity, it appeared protected from hepatic steatosis, hypertriglyceridemia and insulin resistance compared to control mice on a high fat diet. This protective effect of PEA-15 deletion features increased adipocyte size, indicating a greater capacity for lipid storage. We further hypothesized that this increased lipid storage capacity would alter lipid-dependent disease progression. Therefore, we assessed the effect of PEA-15 ablation on lipid plaque development in the Apolipoprotein E (ApoE)-null model of atherosclerosis. PEA-15^−/−^/ApoE^−/−^ mice were protected from lipid plaque formation, compared to ApoE^−/−^ mice. This study reveals, for the first time, that PEA-15 can regulate adipose tissue expansion and ectopic lipid accumulation in obesity, influencing metabolic health and limiting the progression of plaque formation in atherosclerosis.

## Methods

### Animal studies

All experiments were performed in accordance with the United Kingdom Animals Act (Scientific Procedure) of 1986 and performed under a project licence approved by the UK Home Office and University of Aberdeen Ethics Board. This study was carried out in compliance with the ARRIVE guidelines. PEA-15^−/−^ mice were a generous gift from Professor Joe W. Ramos (University of Hawaii, USA). The PEA-15^−/−^ mice were bred on the C57BL/6 J genetic background^18^. Adult C57BL/6 J wild type (WT) mice were purchased from Jackson Laboratories. The PEA-15 KO mice were crossed with ApoE^−/−^ mice (C57BL/6 J genetic background) from Jackson Laboratories to generate PEA-15^−/−^/ApoE^−/−^ mice. Animals were group-housed at the Medical Research Facility, Foresterhill Aberdeen on 12 h cycles of light and dark and at ambient temperature. Male mice were 8 weeks of age at the start of the dietary experiments and weighed weekly. Mice were allowed food and water ad libitum. PEA-15^−/−^ mice or WT mice were fed either standard chow diet (CRM (P) 801,722, Special Diets Service) or high fat diet (HFD) containing 60% fat (Envigo). PEA-15^−/−^/ApoE^−/−^ mice and ApoE^−/−^ mice were fed a high cholesterol/high fat diet (HFHC diet) containing 42% kcal from fat, 0.2% total cholesterol (TD.88137 Envigo). PEA-15^−/−^ mice and WT mice were fed HFD for 8 weeks, the PEA-15^−/−^/ApoE^−/−^ mice and ApoE^−/−^ mice were fed HFHC diet for 16 weeks. Mice were sacrificed by CO_2_ anaesthesia and cervical dislocation. Unless stated otherwise, tissues were rapidly dissected post-mortem, snap-frozen in liquid nitrogen and then stored at -70 °C.

### Histology and imaging

Epididymal white adipose tissue (eWAT) was immersed in 1% paraformaldehyde fixative for 2 h. After fixing, the tissues were wax-embedded (Cellwax Plus paraffin wax, melting point 54–57 °C) using the Citadel 2000 Tissue Processor (ThermoFisher). Sections were cut at 5 μm with 100 μm intervals and dried at 37 °C for 48 h and subsequently stained with H&E (VWR) or immunofluorescent with ERK1/2 antibody mouse anti-ERK1/2 (4696 Cell Signaling Technology) and anti-mouse Alexa Fluor 488 (A11029 ThermoFisher). Tissues were imaged using a light microscope or a laser scanning confocal microscope (Zeiss). All analyses were done using Image J software blinded for treatment or genotype. Adipocyte size was manually measured in 500 adipocytes per animal and intensity of immunofluorescent staining was measured in 20 adipocytes per animal. Liver samples were snap-frozen in liquid nitrogen and cryosections (10 μm, with 50 μm intervals) were stained with 0.2% Oil Red O staining (Sigma-Aldrich), followed by haematoxylin essentially as in^21^. Oil Red O staining was analysed 6 liver sections per animal. Images of *en face* longitudinal opened thoracic aortae were analysed for presence and coverage of plaque area as in^22^.

### Triglyceride assay in serum and liver samples

Blood was collected in SST amber tubes (BD Microtainer) during terminal procedures after 5 h fasting. After 30 min incubation at room temperature, the tubes were spun at 5000×*g* to isolate the serum. Frozen liver tissue samples were homogenized in PBS and centrifuged at 7000×*g* for 15 s at 4 °C. Total triglyceride levels in the serum and liver supernatants were analysed using a Triglyceride Determination Kit (Sigma-Aldrich) as described in^23^. Liver triglyceride levels were expressed as per gram of tissue.

### Cell culture of 3T3-L1 pre-adipocytes

Mouse 3T3-L1 pre-adipocytes (ATCC CL-173) were maintained and subsequently differentiated as previously described in^24^. si-RNA-mediated knockdown was performed on confluent 3T3-L1 pre-adipocytes using Lipofectamine 3000 and Opti-Mem according to the manufactures’ instructions (ThermoFisher). PEA-15 siRNA (sc-37486 Santa Cruz Biotechnology) and non-targeting control siRNA (sc-37007 Santa Cruz Biotechnology) where used at a final concentration of 125 nM in complete medium. Two days post-transfection, the medium was changed to MDI stimulation.

### Immunoblotting

Proteins from eWAT and livers were extracted using RIPA lysis buffer. Protein samples (10 μg) were subjected to 12% SDS-PAGE and transferred to nitrocellulose membrane sheets (GE Healthcare Life Sciences) using standard protocol. Proteins from 3T3-L1 pre-adipocytes were extracted using RIPA lysis buffer (for total extracts) or HEPES/KCl/EDTA buffer containing Complete Protease Inhibitor Cocktail (Roche) (for cytoplasmic and nuclear extracts). Cytoplasmic extracts were obtained by collecting the supernatant after centrifuging at 14,000 rpm for 3 min at 4 °C. The nuclear extracts were obtained by subsequently incubating the pellet in HEPES/NaCl/EDTA/glycerol buffer containing Complete Protease Inhibitor Cocktail for 2 h at 4 °C. The supernatant containing the nuclear extracts were collected after centrifuging at 14,000 rpm for 10 min at 4 °C. Immunoblotting was performed using the following antibodies: rabbit anti-PEA-15, rabbit anti-FABP4/aP2, rabbit anti-ERK1/2, rabbit anti-pERK1/2 rabbit anti-lamin A/C, rabbit anti-GAPDH and anti-βactin (Cell Signaling Technology). Rabbit anti-CD68, mouse anti-PCNA and rabbit anti-RUNX1T1/ETO were from Abcam. Anti-rabbit and anti-mouse secondary antibodies were from Abcam. Immunodetected proteins were visualised using an enhanced chemiluminescence (ECL) kit (Bio-Rad) and quantified by densitometry scanning using Image J software. Where full length immunoblots are not shown in the supplementary data, this is because membranes were cut before incubation with primary antibodies. This was undertaken as it minimised the amount of tissue required for each experiment and reduced the number of animals used in this study.

### Electrophoretic mobility shift assay (EMSA)

EMSA was performed using the LightShift Chemiluminescent EMSA kit (ThermoFisher) per manufacturer’s protocol. Nuclear extracts (2 μg) of si-RNA transfected 3T3-L1 pre-adipocytes were used to incubate with a biotin-labelled probe containing the binding sequence of Elk-1; TTT GCA AAA TGC AGG AAT TGT TTT CAC AGT (5′3′) (ThermoFisher). For competition experiments, an Elk-1 specific, unlabelled cold probe (ThermoFisher) was added in 30 times molar excess. Protein-DNA complexes were resolved on a 6% polyacrylamide gel. Bands were visualized using streptavidin–horseradish peroxidase detection reagent provided by the EMSA kit.

### Proliferation assay

Cell growth was monitored by 3-[4,5-dimethylthiazol-2-yl] 2,5-diphenyl tetrazolium bromide (MTT) assay. In brief, 3T3-L1 pre-adipocytes were seeded into 96-well plates at a density of 2500 cells per well and maintained in complete medium for 48 h. si-RNA-mediated knockdown was performed and cells were incubated for 48 h. Cell growth was determined using the MTT kit (Sigma), following manufactures instructions. The optical density values at 570 nm were recorded using an absorbance detection reader (BMG Labtech).

### Quantitative polymerase chain reaction

Following treatments, RNA was extracted from differentiated 3T3-L1 pre-adipocytes using a RNeasy Mini Kit (Qiagen) according to the manufacturers protocol. RNA samples were DNase treated (Thermo) and cDNA was synthesised using MMLV reverse transcriptase (Promega). Quantitative real-time polymerase chain reaction was performed using a CFX384 qPCR system (Bio-Rad). Power SYBR green detection was measured using the following primers: *pea-15* (FW-5′-GACCAACAACATCACCCTTGA-3′, RV-5′-TCTCCAGGAAGCTAAACCAGG-3′), *ap2* (FW-5′-CAAACTGGGCGTGGAATTCG-3′, RV-5′-ACCAGCTTGTCACCATCTCG-3′), *perilipin* (FW-5′-CACTCTCTGGCCATGTGGAT-3′, RV-5′-AGAGGCTGCCAGGTTGTG-3′), *adiponectin* (FW-5′-TGTTCCTCTTAATCCTGCCCA-3′ 5′-CCAACCTGCACAAGTTCCCTT-3′) and were normalised to the GeoMean of reference genes *ywhaz* (FW-5′-GAAAAGTTCTTGATCCCCA-3′, RV-5′-TGTGACTGGTCCACAATTCCTT-3′), and *nono* (FW-5′-GCCAGAATGAAGGCTTGACTAT-3′, RV-5′-TATCAGGGGGAAGATTGCCCA-3′), using Pfaffl Equation for relative quantification.

### Insulin and glucose tolerance tests (ITT/GTT)

ITT and GTT assays were as described in^21,22^. Prior to GTT, mice were fasted for 5 h. Basal glucose levels were determined by glucometer readings (AlphaTRAK, Zoetis) from tail punctures. Mice were intraperitoneal injected with a 2 mg/g d-glucose (20%, Sigma-Aldrich) bolus. Blood glucose was monitored at 15, 30, 60 and 90 min post-injection. Mice had ad libitum excess to water throughout. ITT was performed by intraperitoneal injection of 0.75 mU/g insulin (Hypurin Bovine Neutral, Wockhardt) per gram of body weight of fasted (5 h) mice.

### Statistical analysis

Statistical analyses were performed using Prism software (GraphPad Software). For single comparisons, unpaired student t test was applied. For multiple comparisons, either one- or two-way analysis of variance followed by Bonferonni post hoc test was used, as appropriate. A value of p < 0.05 was considered significant. Scatter graphs were generated using Prism 9 (GraphPad).

## Results

### PEA-15 expression is increased in adipose tissue following HFD

**Figure 1:**
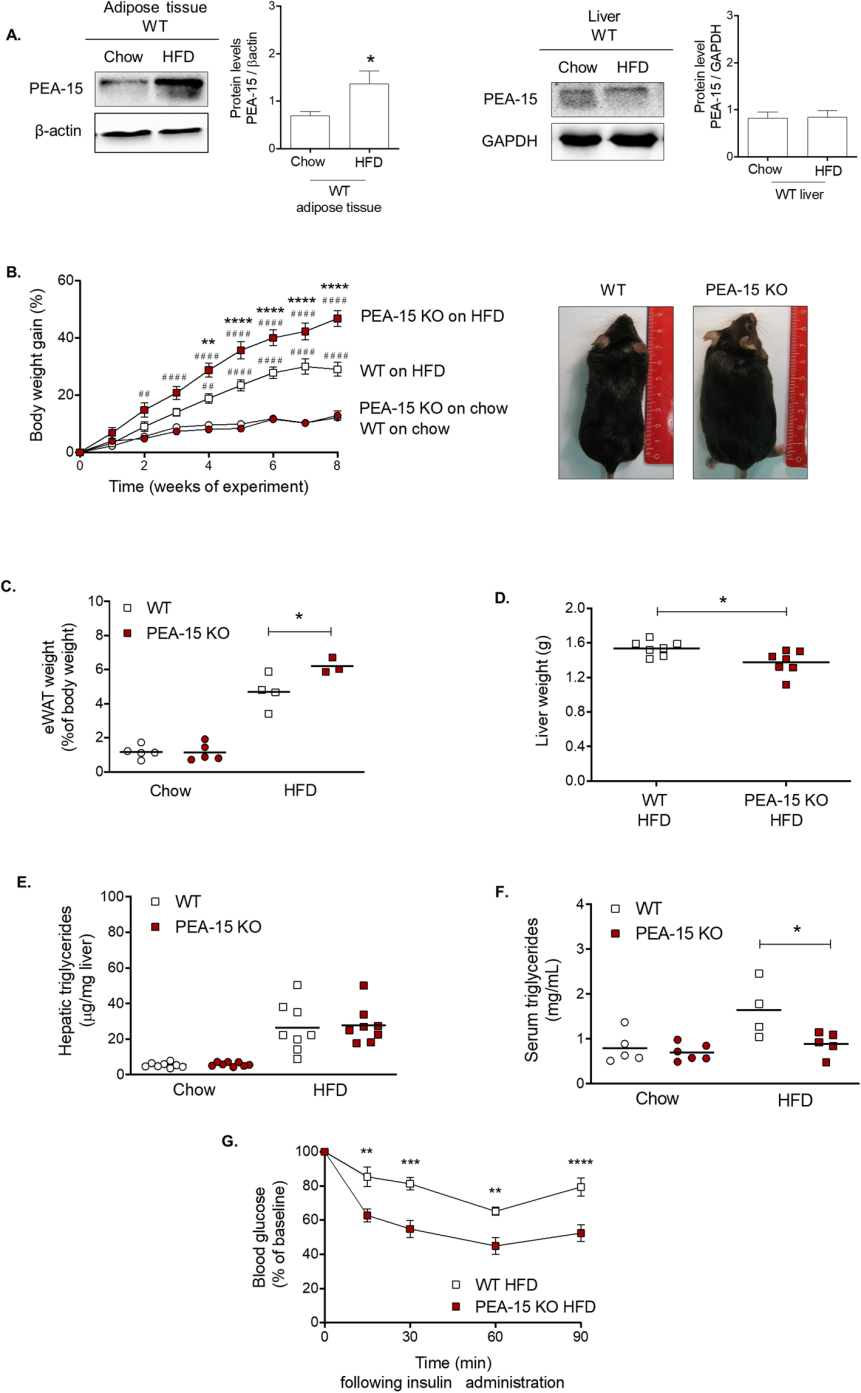
Characterisation of PEA-15 knockout (KO) mice. (**A**) Representative immunoblots for protein levels of PEA-15 in eWAT and liver samples of WT mice after 8 weeks of chow or HFD. Graph displays protein band quantification of PEA-15 as mean ± SEM, expressed as a ratio of β–actin or GAPDH, n = 4–5. *P < 0.05 using Student t test. Full immunoblot images are shown in supplementary Fig. 5. (**B**) Mean body weight gain of PEA-15 KO mice and WT. Gross morphology of WT and PEA-15 KO mice after 8 weeks of HFD. **P < 0.01 and ****p < 0.0001 when comparing genotypes and ##p < 0.01 and ####p < 0.0001 when comparing diets using two-way ANOVA and Bonferroni post hoc test, n = 8. (**C**) Weights of eWAT after 8 weeks of HFD. *P < 0.05 using two-way ANOVA and Bonferroni post hoc test. Significance between WT chow and WT HFD (p < 0.05) is not indicated in graph. (**D**) Livers were weighted directly after dissection after week 8 of the diet intervention, n = 7, *p < 0.05 using Student t test. (**E**) Triglyceride content was measured in liver (n = 8). *P < 0.05 when using two-way ANOVA and Bonferroni post hoc test. Significance between chow and HFD (p < 0.05) is not indicated in graph. (**F**) Triglyceride content was measured in serum (n = 4–6). *P < 0.05 when using two-way ANOVA and Bonferroni post hoc test. Significance between chow and HFD (p < 0.05) is not indicated in graph. (**G**) ITT assay on fasted (5 h) mice after 11 weeks of HFD. Graph shows glucose concentration as the mean ± SEM. **P < 0.01, ***p < 0.001 and **** p < 0.0001 using two-way ANOVA and Bonferroni post hoc test, n = 6–7. Area under the curve (AUC) calculations of graph in G of blood glucose levels. ***P < 0.001 using Student t test, n = 6–7. All graphs were produced using GraphPad Prism version 9.0.0 for Windows, GraphPad Software, San Diego, California USA, www.graphpad.com.

To investigate whether PEA-15 expression could have a role during adipose tissue expansion, we compared PEA-15 protein levels in chow versus HFD-fed mice. PEA-15 protein levels significantly increased following HFD feeding (Fig. 1A) indicating dynamic regulation of PEA-15 during adipose tissue expansion and a possible role in this process. In contrast, the levels of PEA-15 protein in the liver were unchanged following HFD (Fig. 1A), indicating selective regulation of PEA-15 may occur in adipocytes.

### PEA-15^−/−^ mice on a high fat diet had increased adiposity but were protected from several features of metabolic disease

We next examined the role of PEA in diet induced adipose expansion and metabolic health in obesity. PEA-15^−/−^ mice were fed either chow or HFD diet for 8 weeks. No significant differences in body weight were observed between PEA-15^−/−^ and WT prior to dietary manipulation (Fig. 1). Body weight gain of PEA-15^−/−^ mice on chow diet was similar to WT mice. When challenged with HFD for 8 weeks, PEA-15^−/−^ mice exhibited a significantly increased body weight gain of approximately 50% compared to the HFD-fed WT mice (Fig. 1B). This was also the case when total body weight was compared (supplementary Fig. 1A). This increased weight gain compared to the WT mice was significant from week 4 of HFD-feeding onwards. In, addition, PEA-15^−/−^ mice on HFD had a significantly greater mass of eWAT in the PEA-15^−/−^ mice on HFD (Fig. 1C). In contrast, the weight of the liver in the PEA-15^−/−^ mice on HFD was significantly lower compared to the WT mice on HFD (Fig. 1D).

Increased adiposity is normally characterized by increased release of free fatty acids in the circulation which are taken up by the liver to form triglycerides and phospholipids^2,3^. Triglyceride content in the liver was significantly increased in HFD mice compared to chow-fed mice as expected (Fig. 1E). However, despite the increased adiposity of the PEA-15^−/−^ mice, triglyceride content was not significantly different between PEA-15^−/−^ mice and WT mice. This was confirmed by Oil Red O staining of the liver cross sections, which showed no increased hepatic lipid accumulation in the PEA-15^−/−^ mice on HFD (supplementary Fig. 2). Interestingly, despite their increased weight gain and adiposity, serum triglyceride levels were significantly lower in HFD-fed PEA-15^−/−^ than HFD-fed WT mice and instead remained similar to levels observed in chow fed mice (Fig. 1F).

**Figure 2:**
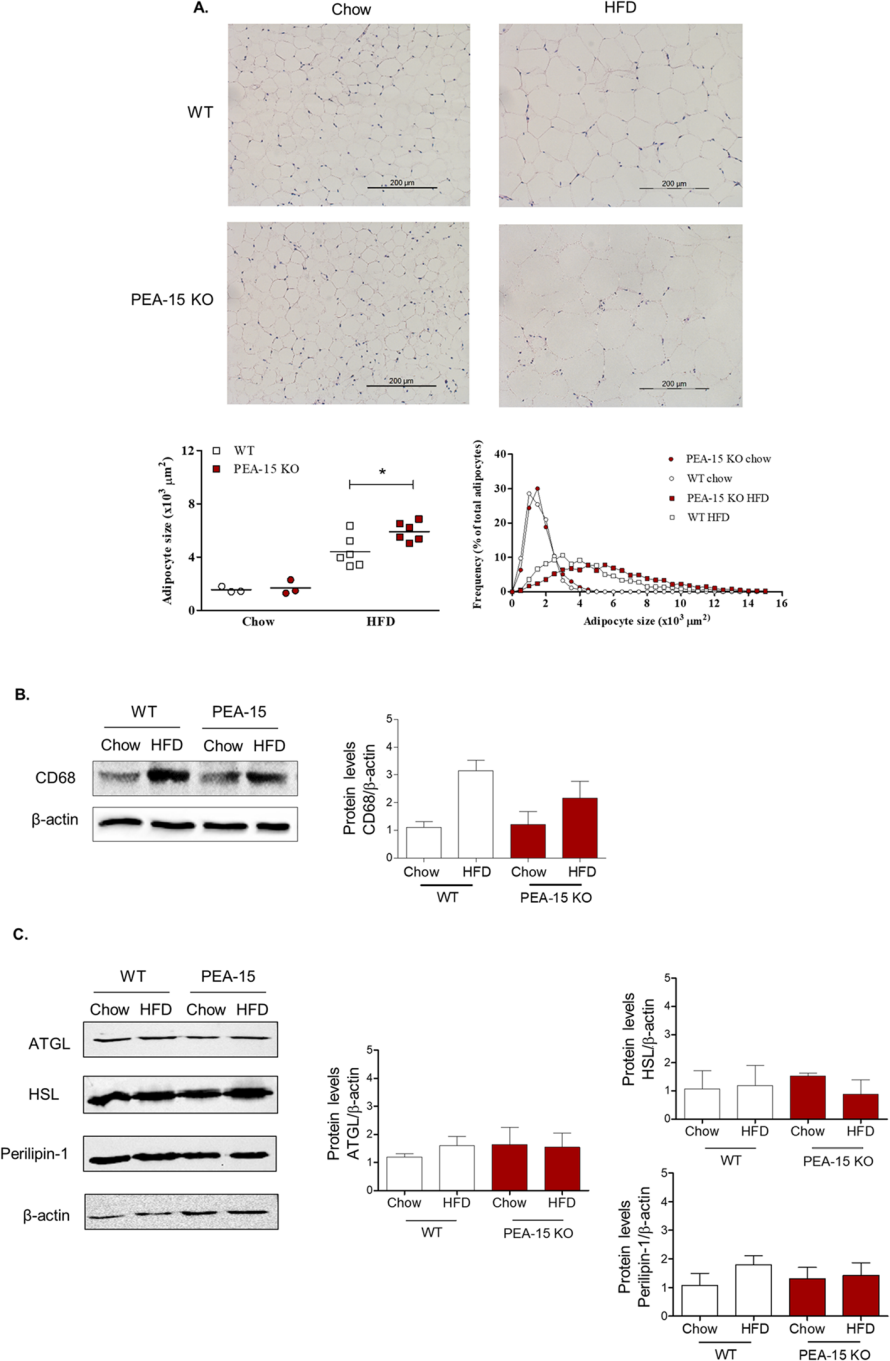
Phenotype of visceral adipose tissue of PEA-15 knockout (KO) mice. (**A**) Representative bright field images of H&E stained eWAT of PEA-15 KO mice and WT mice after 8 weeks of chow or HFD. Graphs display quantification of adipocyte size and size frequency using the H&E stained images. *P < 0.05 using two-way ANOVA and Bonferroni post hoc test. Significance between chow and HFD (p < 0.05) is not indicated in graph. (**B**) Representative immunoblots for eWAT protein levels of CD68 (marker for tissue-infiltrated macrophages). Full immunoblot images are shown in supplementary Fig. 6. Graph displays protein band quantification mean ± SEM, expressed as a ratio of β–actin, n = 4–5. (**C**) Representative immunoblots for eWAT protein levels for markers of lipid metabolism: adipose triglyceride lipase (ATGL), hormone-sensitive lipase (HSL) and perilipin-1. Full immunoblot images are shown in supplementary Fig. 7. Graph displays protein band quantification mean ± SEM, expressed as a ratio of β–actin, n = 4.

We also examined the effect of PEA-15 loss on insulin sensitivity by performing an insulin tolerance test (ITT). Following HFD-feeding for 11 weeks PEA-15^−/−^ mice displayed significantly improved responses to insulin injection compared with WT mice (Fig. 1G). We observed no differences in fasting serum glucose levels between PEA-15^−/−^ mice and WT mice on either chow or HFD (supplementary Fig. 3A). In addition, no differences in glucose tolerance were observed between chow-fed PEA-15^−/−^ mice and WT mice, however HFD-fed PEA-15^−/−^ mice displayed temporary glucose intolerance at 60 min, which rapidly returned to the same level as WT mice by 90 min, indicating that PEA-15^−/−^ mice on HFD are mildly glucose intolerant (supplementary Fig. 3B).

**Figure 3:**
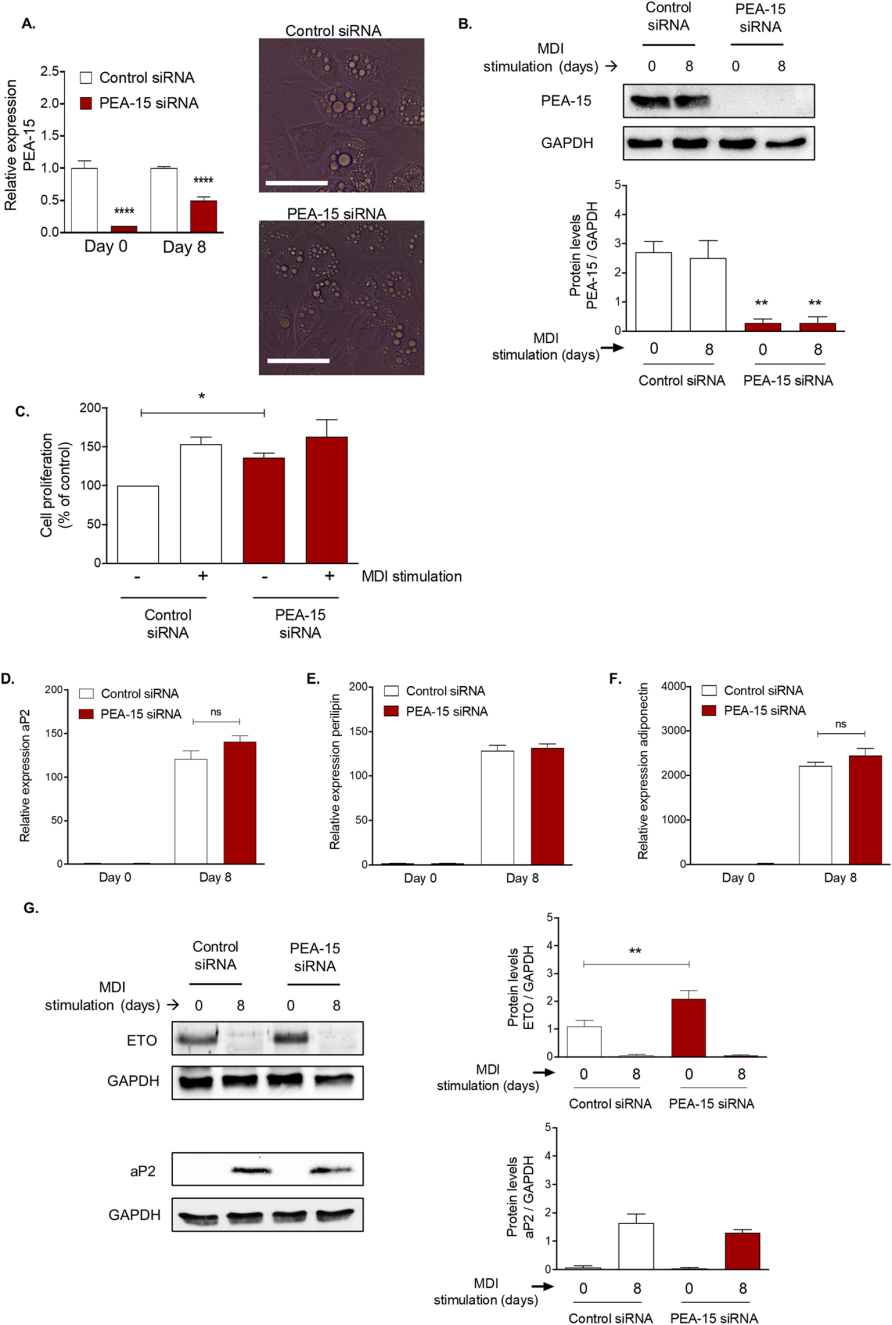
Inhibition of PEA-15 in pre-adipocytes increases proliferation but does not significantly affect adipocyte differentiation. 3T3-L1 pre-adipocytes were transfected to knockdown PEA-15 using PEA-15 siRNA or non-targeting siRNA (control). After 48 h the cells were either harvested or stimulated to differentiate for 8 days. (**A**) Relative gene expression of PEA-15 in 3T3-L1 pre-adipocytes is expressed as mean ± SEM relative to the geomean of Nono and ywhaz expression. ****P < 0.0001 using two-way ANOVA and Bonferroni post hoc test, n = 4. Representative bright field images of 3T3-L1 cells demonstrating well-developed lipid droplets in cells transfected with either PEA-15 siRNA or control siRNA. Scale bar represents 100 μm. (**B**) Representative immunoblots for PEA-15 and GAPDH are shown and graph shows protein band quantification as mean ± SEM, expressed as a ratio of GAPDH, n = 3. Full immunoblot images are shown in supplementary Fig. 8. (**C**) 48 h post-transfection, 3T3-L1 pre-adipocytes were subjected to the proliferation assay (MTT), either unstimulated or stimulated with MDI for 24 h. Data expressed as mean ± SEM. *P < 0.05 using two-way ANOVA and Bonferroni post hoc test, n = 4. (**D**) Relative gene expression of aP2, perilipin1 (**E**) and adiponectin (**F**) in 3T3-L1 pre-adipocytes is expressed as mean ± SEM relative to the geomean of nono and ywhaz expression, n = 4. Significance between day 0 and day 8 (p < 0.01) is not indicated in graph. (G) Representative immunoblots for protein levels of ETO (marker for early adipocyte proliferation), aP2 (marker for late adipocyte development) and GAPDH in transfected 3T3-L1 pre-adipocytes are shown. Full immunoblot images are shown in supplementary Fig. 9. Graph displays protein band quantification as mean ± SEM, expressed as a ratio of GAPDH, n = 3. Significance between day 0 and day 8 (p < 0.01) is not indicated in graph. *P < 0.05 and **p < 0.01 using two-way ANOVA and Bonferroni post hoc test.

### Adipocyte cell size is increased in eWAT from PEA-15^−/−^ mice

The observed increase in adiposity of PEA-15^−/−^ mice on HFD coupled with decreased plasma triglyceride levels suggested an increased efficiency in lipid storage. We therefore examined the effect of PEA-15 loss in adipose tissue in more detail.

We first examined the adipocyte cell size in sections of eWAT. Mice on chow diet showed no difference in adipocyte size between the WT mice and PEA-15^−/−^ mice, which was unchanged over the course of the experiment (Fig. 2A). Interestingly, PEA-15^−/−^ mice on HFD had significantly larger adipocytes (approximately 34% increase) after 8 weeks compared to the WT mice on HFD, as seen as a shift in size distribution (Fig. 2A).

Increased adiposity and adipocyte hypertrophy are typically associated with chronic low-grade inflammation^2,5,6^. Consistent with this, macrophage tissue-infiltration in eWAT, as measured by the protein levels of CD68, was increased by HFD-feeding for 8 weeks in WT mice (Fig. 2B). However, despite having larger adipocytes, CD68 expression in the eWAT of PEA-15^−/−^ mice failed to show the same increase as a result of HFD-feeding and was not significantly changed from levels in chow fed PEA-15^−/−^ mice. In addition, we examined markers of lipid metabolism including adipose triglyceride lipase (ATGL), hormone-sensitive lipase (HSL) and perilipin-1 (Fig. 2C) in eWAT form PEA^−/−^ mice. Expression of each of these markers was unchanged, both during HFD and in combination with PEA deletion. Overall these data imply that increased adiposity and adipocyte hypertrophy in the adipose tissue of PEA-15^−/−^ mice is not associated with the negative metabolic effects typically observed with these changes^25^.

### PEA-15 loss in pre-adipocytes does not appear to significantly alter adipogenesis

The increased ability to store dietary lipids in the adipose tissue through increased adipocyte size of the PEA-15^−/−^ mice could indicate a molecular role for PEA-15 in adipocyte differentiation.

To examine this possibility, we next inhibited PEA-15 expression using siRNA in the well-characterised 3T3-L1 pre-adipocyte cell model. Transfection of 3T3-L1 preadipocytes with siRNA targeting PEA-15 prior to the induction of differentiation and during the early stages of adipogenesis was sufficient to cause a robust and sustained inhibition of PEA-15 mRNA and protein expression (Fig. 3A, B). Differentiation of these cells resulted in well-defined lipid droplets in both control siRNA and PEA-15 siRNA-treated cells (Fig. 3A). Knockdown of PEA-15 expression led to a modest but significant increase in the rate of proliferation of preadipocytes (Fig. 3C). However, we did not observe a significant alteration in proliferation specifically associated with the induction of differentiation in these cells.

When cells transfected with either control siRNA or siRNA targeting PEA-15 were induced to differentiate for 8 days, PEA-15 knockdown did not significantly affect the induction of mRNA encoding the mature adipocyte markers aP2, perilipin 1 and adiponectin (Fig. 3D–F). In addition, we examined expression of the preadipocyte marker protein ETO and aP2 protein during adipogenesis^24^. Both these proteins were used as marker proteins of differentiation and neither has any known influence on PEA-15-dependent mechanisms. Immunoblotting revealed that PEA-15 loss did not prevent the dramatic reduction ETO, nor the induction of aP2 protein (Fig. 3G). However, we did observe a modest but significant induction of ETO expression in pre-adipocytes lacking PEA-15, versus those transfected with control siRNA, prior to the induction of differentiation (Fig. 3G).

Overall these data indicate that a decrease in PEA-15 expression may lead to a modest increase in proliferation of unstimulated pre-adipocytes but that PEA-15 loss does not substantially affect adipocyte differentiation per se.

### PEA-15 loss significantly alters ERK signalling and localisation in developing and mature adipocytes

Next, we examined whether altering PEA-15 expression affects ERK signalling in developing 3T3-L1 preadipocytes. Previous studies, including our own, have demonstrated that PEA-15 is bound to, and therefore sequesters, ERK1/2 in the cytoplasm, thereby acting as a cytoplasmic anchor^26,27^. In resting conditions, PEA-15 prevents ERK1/2 translocation to the nucleus and therefore limits ERK1/2-mediated gene transcription^15,18^. Thus, dynamic expression of PEA-15 that alters the ratio of PEA-15:ERK1/2 is important in regulating the level of ERK1/2 nuclear localization^18^. As ERK is a key kinase affecting multiple cellular processes, this offers a potential mechanism whereby PEA-15 could influence adipocyte function.

**Figure 4:**
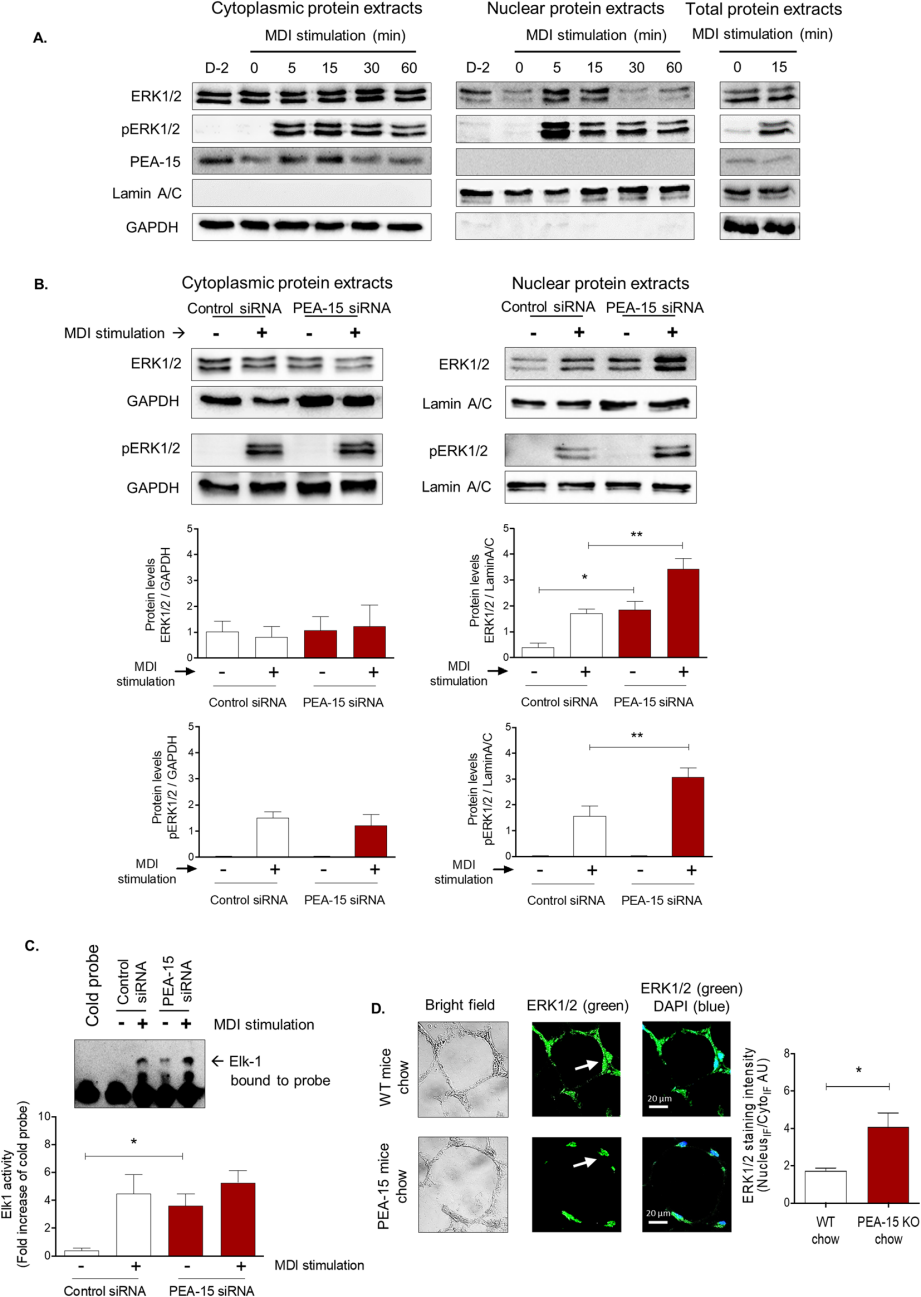
Loss of PEA-15 alters the subcellular localisation and function of ERK1/2 in cultured cells and in vivo. (**A**) Representative immunoblots for PEA-15, total ERK1/2, phosphorylated (p)ERK1/2, lamin A/C (loading control for the nuclear protein extract) and GAPDH (loading control for the cytoplasmic protein extract) are shown. Full immunoblot images are shown in supplementary Fig. 10. Graphs display protein band quantification as mean ± SEM, expressed as a ratio of lamin A/C or GAPDH, n = 3. D-2 means confluent cells, 48 h pre-stimulation. *P < 0.05 and **p < 0.01 when comparing to 0 min using two-way ANOVA and Bonferroni post hoc test and Student t test. (**B**) After 48 h of transfection, 3T3-L1 pre-adipocytes were stimulated with MDI for 15 min. Representative immunoblots for PEA-15, total ERK1/2, phosphorylated (p)ERK1/2, lamin A/C and GAPDH. Full immunoblot images are shown in supplementary Fig. 11. Graphs display protein band quantification as mean ± SEM, expressed as a ratio of lamin A/C or GAPDH, n = 3. Significance (p < 0.05) between stimulated and unstimulated is not indicated in graph. Significant difference from values in control siRNA cells is indicated by *P < 0.05 and **p < 0.01 using two-way ANOVA and Bonferroni post hoc test. (**C**) EMSA to measure Elk-1 activity in transfected 3T3-L1 pre-adipocytes. 48 h post-transfection, the cells were stimulated with MDI for 15 min. Arrow on the representative image denotes a gel shift indicating complex formation of Elk-1 with its DNA binding site. Graph displays mean ± SEM band intensity as a fold over density of cold probe band, n = 3. Significance (p < 0.05) between stimulated and unstimulated control cells is not indicated in graph. Significant difference from values in control siRNA cells is indicated by *P < 0.05 using two-way ANOVA and Bonferroni post hoc test. (**D**) Representative immunofluorescent images of eWAT cross sections of PEA-15 knockout (KO) mice and WT mice on chow diet. Bright field images (left) and immunofluorescent images of immunostained for ERK1/2 (green) and overlaid (right) with the nuclear stain DAPI (blue). Scale bar 20 μm, arrows indicate colocalization of ERK1/2 staining with the DAPI nuclear staining. Graph displays nuclear:cytoplasmic ratio (mean ± SEM) calculated using confocal images analyses comparing eWAT from 4 mice. Significant difference is indicated by *P < 0.05 using Student t test.

First, we confirmed the activation and translocation of ERK1/2 in preadipocytes following stimulation with MDI medium (Fig. 4A). Upon MDI stimulation, the early differentiation of 3T3-L1 cells revealed a rapid and transient ERK1/2 translocation to the nucleus with levels returning to basal levels within 30 min. In addition, there was a concomitant increase in activated (phosphorylated) ERK1/2 (pERK1/2) in the nucleus within 5 min of stimulation which was maintained for at least 60 min. In both resting conditions, and throughout stimulation, PEA-15 was almost exclusively situated in the cytoplasm and not in the nuclear fraction (Fig. 4A). Total cytoplasmic levels of PEA-15 and ERK1/2 proteins did not significantly change following MDI stimulation.

After establishing the nuclear translocation of ERK1/2 in 3T3-L1 pre-adipocytes, we examined ERK1/2 localization following knockdown of PEA-15 as previously described (see Fig. 3). Nuclear localisation of ERK was significantly increased in unstimulated cells in response to PEA-15 knockdown (Fig. 4B). MDI stimulation led to increased nuclear localisation of ERK1/2 in both control and PEA-15 knockdown cells. However, the levels of nuclear ERK1/2 expression remained significantly higher in PEA-15 knockdown cells following MDI treatment. A similar effect was observed when measuring activated, phosphorylated ERK1/2 levels, which were increased in the nucleus of control and PEA-15 knockdown cells in response to MDI treatment but significantly higher in the PEA-15 knockdown preadipocytes. Consistent with the majority of ERK1/2 remaining in the cytoplasm in all cells, we observed no significant effect of PEA-15 knockdown on the levels of cytoplasmic total or phosphorylated ERK1/2, although the induction of ERK1/2 phosphorylation in response to MDI was clearly evident (Fig. 4B).

We next examined whether the effect of PEA-15 loss could significantly alter ERK/2 function in 3T3-L1 pre-adipocytes. Upon release from PEA-15, activated ERK1/2 translocates to the nucleus and directly activates the transcription activator Ets-like protein-1 (Elk-1)^28^. Activation of Elk-1 leads to binding with its specific DNA target sequence and is known to be important in adipocyte differentiation and function^29,30^. Using the Electrophoretic Mobility Shift Assay (EMSA), we measured Elk-1 protein binding to the DNA sequence of the Elk-1 binding element. As expected, Elk-1 activation was increased after MDI stimulation in cells transfected with control siRNA (Fig. 4C). Importantly, knockdown of PEA-15 in 3T3-L1 led to significantly increased Elk-1 activation in the absence of MDI stimulation. Indeed, Elk-1 activation in these cells was similar to that in control cells treated with MDI and did not significantly increase further with MDI stimulation. This indicates that Elk-1 activation is continuously maintained in the absence of PEA-15, presumably due to the constant presence of ERK1/2 in the nucleus.

To determine if the effects observed in 3T3-L1 preadipocyte model are relevant to our in vivo model of PEA-15 deficiency, we compared ERK1/2 localisation in adipocytes in eWAT of WT and PEA-15^−/−^ mice. Immunofluorescent analysis revealed that in adipocytes of the WT mice ERK1/2 staining could be found in both the cytoplasm and in the nucleus (Fig. 4D). In contrast, ERK1/2 was predominantly localized to the nucleus in adipocytes of PEA-15^−/−^ mice, evident from co-localization with DAPI nuclear staining. Consistent with this, quantification confirmed a significantly higher level of nuclear ERK1/2 staining in the adipose tissue of PEA-15^−/−^ (Fig. 4D). Overall these data show that PEA-15 expression can significantly alter ERK1/2 localisation and function in developing and mature adipocytes in culture and in vivo.

### PEA-15^−/−^ improves metabolic health and decreases plaque formation in a mouse model of atherosclerosis

**Figure 5:**
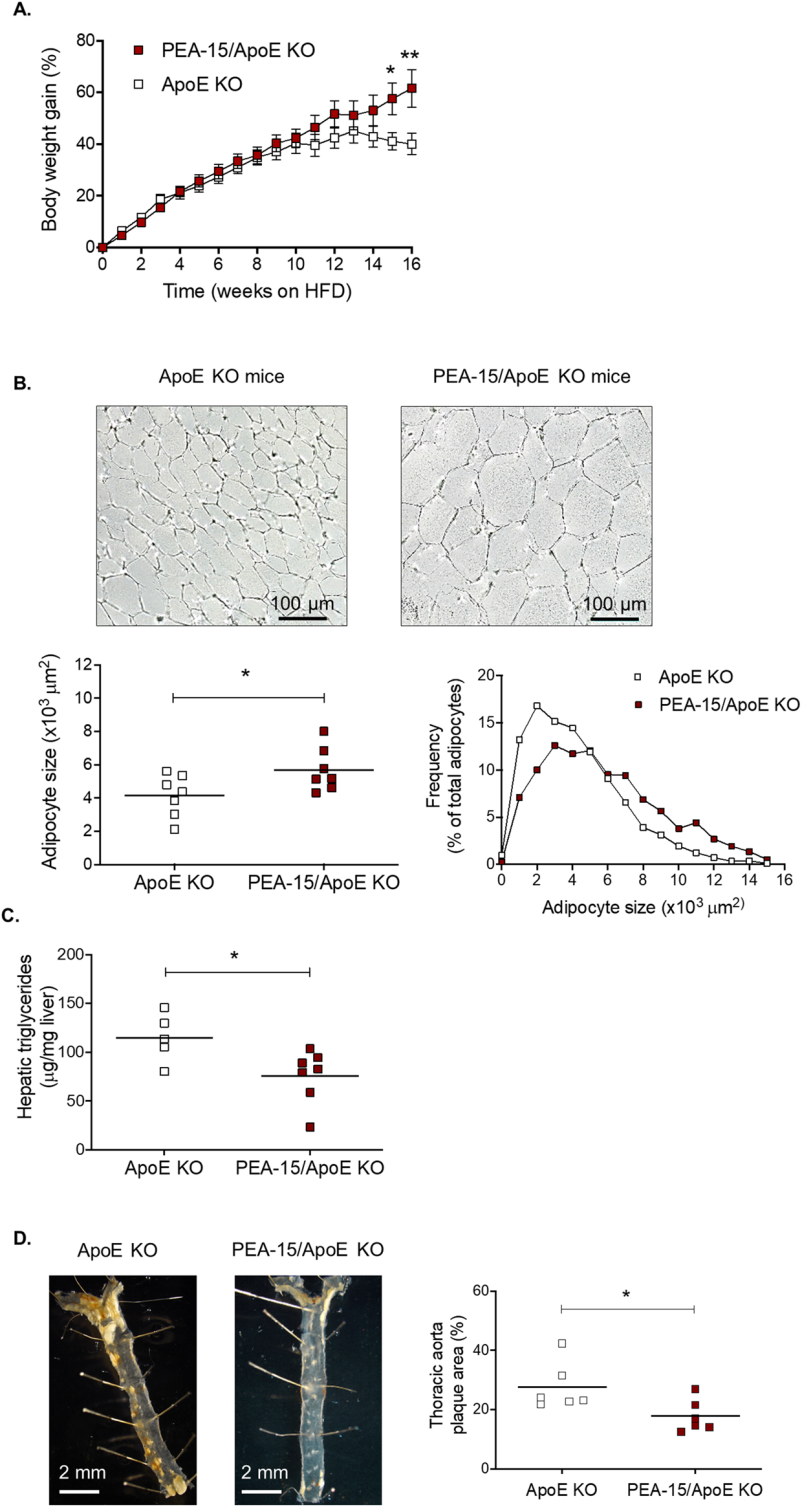
Characterisation of PEA-15/ApoE knockout (KO) mice. (**A**) Body weight gain of ApoE KO mice (n = 7) and PEA-15/ApoE KO mice (n = 11) during the period of the HFHC diet. Graph displays body weight gain as percentage of start weight before experiment. All data are presented as the mean ± SEM. Significant difference between the 2 groups is indicated by *P < 0.05 and **p < 0.01 using two-way ANOVA and Bonferroni post hoc test. (**B**) Representative bright field images of cross-sections of unstained eWAT. Graph displays adipocyte size measurements, significant difference between ApoE KO and PEA-15/ApoE KO is indicated by *p < 0.05 using Student t test, n = 7. (**C**) Liver triglyceride levels in ApoE KO mice (n = 5) and PEA-15/ApoE KO mice (n = 7) measured in duplicate and expressed as μg per mg of lysed liver tissue, *p < 0.05 using Student t test. (**D**) Representative *en face* bright field images of the thoracic aortae. Graph displays the analysis of plaque area, expressed as the percentage of the aortic surface covered by lesions (yellow formations). Significant difference between ApoE KO and PEA-15/ApoE KO is indicated by *p < 0.05 using Student t test, n = 6. All data analysis and figures were produced using GraphPad Prism version 9.0.0 for Windows, GraphPad Software, San Diego, California USA, www.graphpad.com.

Our earlier results showed that PEA-15 could play a key role in the regulation of lipid metabolism by altering the storage capacity of adipose tissue. This could reduce lipid overflow to other organs such as the liver and may explain reduced plasma triglycerides and improved insulin sensitivity in PEA-15^−/−^ mice. Another key effect of elevated circulating lipids in obesity is the development of atherosclerosis. Thus, we next wished to determine whether PEA-15 loss could be protective against atherosclerotic plaque formation. To examine this, we used the well-established mouse model of atherosclerosis ApoE^−/−^, and crossed these to the PEA-15^−/−^ mice to ApoE^−/−^/PEA-15^−/−^ mice. Mice were fed a HFHC diet for 16 weeks to induce plaque development^22^. As previously seen with HFD-fed PEA-15^−/−^ mice (Fig. 1B), the ApoE^−/−^/PEA-15^−/−^ mice also had a significantly increased weight gain compared to ApoE^−/−^ mice following HFHC diet-feeding, although this was only evident after 15 weeks of dietary challenge (Fig. 5A). We observed no significant differences between control ApoE^−/−^ and ApoE^−/−^/PEA-15^−/−^ mice prior to HFHC diet-feeding (supplementary Fig. 11B).

We next examined whether adipocyte size was also affected by PEA-15 loss in ApoE^−/−^ mice. As in HFD diet-fed PEA-15^−/−^ mice, adipocytes in the eWAT of ApoE^−/−^/PEA-15^−/−^ mice were increased in size compared to ApoE^−/−^ mice following HFHC diet-feeding (Fig. 5B). Quantification of the adipocyte size distribution in ApoE^−/−^ versus ApoE^−/−^/PEA-15^−/−^ mice confirmed a shift to larger adipocytes with the loss of PEA-15 (Fig. 5B).

To determine whether alterations observed to adipose tissue might impact upon lipid accumulation in the liver, we analysed hepatic triglyceride content in HFHC diet-fed ApoE^−/−^ and ApoE^−/−^/PEA-15^−/−^ mice. Hepatic triglyceride content was significantly lower in the ApoE^−/−^/PEA-15^−/−^ mice (Fig. 5C). Lower lipid accumulation in the liver was also confirmed by Oil Red O staining of the liver cross sections (supplementary Fig. 4).

Finally, thoracic aortae from ApoE^−/−^ and ApoE^−/−^/PEA-15^−/−^ mice were examined following 16 weeks of HFHC diet-feeding. At this time point, advanced atherosclerotic plaque formation was clearly visible in the aortae of ApoE^−/−^ mice (Fig. 5D). However, although some plaque formation was observed in the aortae of ApoE^−/−^/PEA-15^−/−^ mice, the area covered by plaques was significantly reduced compared to that in ApoE^−/−^ mice.

Overall, our data strongly imply that the loss of PEA-15 increases the storage capacity of adipocytes in WAT. As a result, the ApoE^−/−^/PEA-15^−/−^ mice were capable of more efficiently buffering the excessive lipid intake, protecting against ectopic lipid accumulation in the liver and the aorta, thereby slowing lipid plaque formation.

## Discussion

In the current study, we demonstrate for the first time that PEA-15 plays a critical role in the regulation of adipocyte lipid storage. Adipocytes from mice lacking PEA-15 globally are significantly larger than those in WT mice following over-nutrition in two different in vivo models. Unusually, this is not associated with increased inflammation, hepatic steatosis and insulin resistance and offers protection from severe atherosclerosis on an the ApoE-null background. Thus, we propose that lipid overflow from WAT is significantly reduced in PEA-15^−/−^ mice, due to an enhanced adipocyte expansion capacity and that this prevents ectopic lipid accumulation in other tissues and protects against atherosclerosis. This strongly implies that PEA-15 could be a gatekeeper for safe adipocyte expansion and lipid storage. As such it may offer a novel and important target to improve metabolic health by promoting healthy adipose expansion.

Although the phenomenon of pathogenic lipid overflow from adipose tissue in obesity is well-studied, the physiological regulation of adipocyte lipid storage capacity remains poorly understood^31^. Our initial observation that PEA-15^−/−^ mice on HFD have increased adiposity but unchanged liver triglyceride and decreased plasma triglycerides reveals a distinct and novel metabolically protected phenotype. This is similar to previously reported leptin-deficient ob/ob mice overexpressing adiponectin wherein facilitated expansion of adipose tissue protected against lipid overflow into the liver^32^. It is well known that restricting adipose tissue expansion and thereby safe lipid storage capacity, as occurs in syndromes of lipodystrophy, accelerates the detrimental effects of excessive lipid intake^1,6^. Evidence that PEA-15 is part of the physiological regulation of adipocyte lipid storage capacity is provided by our observation that the expression of PEA-15 in adipose tissue is dynamic during HFD induced adipose expansion in WT mice. Adipocyte PEA-15 expression is elevated following weight gain both in our study and previously published work^21^. We propose that PEA-15 induction plays a key role in the physiological mechanisms limiting adipocyte expansion, such that when PEA-15 is absent, this brake is removed and adipocytes can enlarge further without inducing detrimental metabolic consequences.

In the present study the deletion of PEA-15 led to an enhanced glucose lowering effect in an insulin tolerance test performed on obese mice following high fat diet feeding. Overall, this is consistent with previous studies showing that, conversely, global over-expression of PEA-15 in mice can induce a type 2 diabetic phenotype, even on a chow diet^33^. However, despite improved insulin tolerance, our HFD-fed PEA-15^−/−^ mice displayed modestly reduced glucose tolerance. We speculate that altered substrate utilisation in PEA-15^−/−^ mice, possibly adapted to favour lipid disposal, may render these mice both more responsive to the acute actions of exogenous insulin and yet less rapidly able to dispose of a discrete bolus of glucose. This switch to greater lipid use would be consistent with both the enlarged adipocytes, lower hepatic steatosis and protection from atherosclerotic plaque formation. Alternatively, if endogenous insulin production is reduced, but insulin sensitivity increased in PEA-15^−/−^ mice, this could also result in modest glucose intolerance despite apparent improved insulin sensitivity. Further work is required to fully understand this phenomenon.

In PEA-15^−/−^ mice, although we cannot exclusively conclude that all the weight gain on HFD is directly a result of an increased fat mass, the increased adipose tissue weight following HFD (approximately 30% increase compared to wild type eWAT) correlates with the body weight gain (similarly approximately 30%). While we do not yet know the relative contributions of increased calorie intake versus reduced energy expenditure in the increased weight gain observed with PEA-15 deletion, the adipose tissue expansion and increased lipid storage is at least partly due to the increased cell size of adipocytes. Larger adipocytes typically correlate with impaired metabolic regulation^34^ and have also been associated with increased inflammation^35^. In contrast, in our study this correlation does not hold as adipocytes from PEA-15^−/−^ mice are not linked with either a detrimental metabolic phenotype or increased inflammation of WAT. The ability of PEA-15 inhibition to increased cell size and yet exert a metabolically protective effect suggests that targeting PEA-15 offers a means to enhance the safe lipid storage capacity of adipocytes. Whilst the risk of metabolic disease increases with weight gain, the threshold at which this causes metabolic disease varies significantly between individuals, dependent on multiple factors including adipose tissue distribution, genetics, sex and ethnicity^6^. The contribution of PEA-15 expression to this phenomenon is unknown but clearly worthy of future study. Although our evidence indicates that adipocytes are likely to have a role in PEA-15-dependent effects on lipid storage, at this stage we cannot rule out the involvement of other tissues, including skeletal muscle^20^. This will require further investigation using tissue-specific deletion of PEA-15. Regardless, if it were possible to manipulate PEA-15 levels or function therapeutically, this could offer a means to improve safe lipid storage and thereby improve metabolic health at any given BMI^32^.

A key remaining question is the molecular mechanisms via which PEA-15 influences adipocyte size and how its loss improves metabolic health. We did not observe any major changes in adipocyte differentiation in 3T3-L1 preadipocytes with decreased PEA-15 expression. However, it is possible that PEA-15 loss differentially affects adipocyte development of adipocytes from different stem cell populations such that adipocytes in PEA-15^−/−^ mice are of a type inherently better able to safely store lipids. For example, expansion of subcutaneous adipose tissue is typically associated with a better metabolic profile than expansion of visceral depots^1^. Subcutaneous adipocytes are usually larger than those in visceral depots^36^, whilst obese individuals with diabetes may have smaller subcutaneous adipocytes than non-diabetic obese individuals implying the ability to enlarge adipocytes in this depot could be protective^37^. Thus, if PEA-15 loss drives a switch in visceral adipocytes to a phenotype more characteristic of subcutaneous adipocytes, this could explain the protective phenotype we observe in our PEA-15^−/−^ mice. Alternatively, PEA-15 loss may affect the control of lipogenesis and/or lipolysis, the secretion of adipokines or inflammatory cytokines, cell senescence, the cytoskeleton of the adipocyte or its interaction with the extracellular matrix and the vasculature. All have the capacity to influence whether expansion of adipose tissue occurs in a metabolically healthy way^1^ and offer avenues for future investigations of PEA-15 function in adipose tissue^1,2,38^.

We observed increased nuclear localization of ERK1/2 in WAT from PEA-15^−/−^ mice, in agreement with other cell types where PEA-15 expression is decreased^18,39^. ERK1/2 has been previously demonstrated to have a critical role in adipocyte response to adipose tissue expansion^40^. We also demonstrate that ERK1/2 translocates to the nucleus following activation in 3T3-L1 pre-adipocytes as expected and this balance is altered when PEA-15 expression is knocked down, favouring nuclear localization even in unstimulated cells. Importantly, we reveal that downstream Elk-1 transcription is activated but only under resting conditions in these cells, highlighting that manipulating PEA-15, and thereby ERK1/2 intracellular localization, have potential consequences for gene expression. This correlates with a small increase in proliferation in undifferentiated cells in vitro. It is not clear how this observed increase in proliferative signalling in undifferentiated cells in vitro translates in vivo. Previous studies have shown that Elk-1 can influence adipocyte function and may be important for some cellular responses to FGF21 and insulin which are impaired in obesity^29,30,41^. As FGF-21 stimulates ERK activity and has adipocyte-dependent beneficial effects on insulin sensitivity^6^, it will be interesting to examine whether PEA-15 loss preserves FGF-21 responses in the face of obesity as part of an improved metabolic profile in our mice^41^. Conversely, chronically increased cytosolic ERK activity has recently been shown to occur in obesity and lead to enhanced lipolysis, whilst ERK inhibition reduces lipolysis and improves insulin sensitivity in vivo^42^. Increased PEA-15, as we observe in obese mice, favours cytosolic ERK accumulation. Hence, it is possible PEA-15 can influence lipolysis and insulin sensitivity via this mechanism.

Uncovering the pathogenesis of arterial lipid plaque and its relationship to obesity is critical to fully understanding atherosclerosis-dependent morbidity and mortality^9^. While it is likely that lipid overflow is closely associated with increased circulating plasma lipids which underlie the pathogenesis of atherosclerotic plaque development, the relationship to lipid storage is not clear. Our novel mouse model, PEA-15^−/−^ mice crossed with the well-characterised atherosclerotic mouse model, on HFHC revealed an atheroprotective effect in these mice. Decreased lipid plaque development was paralleled by a decrease in hepatic triglyceride levels and an increased adipocyte cell size. Although we did not measure plasma lipoproteins directly, our previous mouse study has indicated that changes in triglycerides are closely mirrored in lipoprotein levels^22^. It is therefore likely that observed differences in PEA-15^−/−^ are not the result of changes to triglycerides or cholesterol. The protective effect of PEA-15^−/−^ was therefore maintained in the PEA-15^−/−^/ApoE^−/−^ mouse model and translated into protection in atherosclerosis. This study therefore provides further evidence for a direct link between atherosclerosis and lipid storage capacity, and support for the notion that altering adipose tissue function could be a therapeutic approach in this disease^6,43^.

In summary, this study has revealed for the first time that the cytoplasmic protein PEA-15 is a novel regulator of adipose tissue expansion and plays a role in the regulation of adipocyte size. Decreased PEA-15 expression results in a metabolically improved phenotype, likely driving decreased lipid overflow from adipose tissue. Importantly, these effects of PEA-15 inhibition on adipose tissue can decrease progression of the lipid-related cardiovascular disease, atherosclerosis.

## Supplementary Information

Supplementary Information.
