# Supplementary material for: Phosphoprotein enriched in astrocytes (PEA)-15 is a novel regulator of adipose tissue expansion
Source: Sci Rep. 2021 Mar 26;11:6949. doi: 10.1038/s41598-021-86250-x (PMC7997924; doi:10.1038/s41598-021-86250-x)
Supplement: Supplementary file 1 — Supplementary Information. [file 41598_2021_86250_MOESM1_ESM.pdf]

SUPPLEMENTARY DATA

Phosphoprotein enriched in astrocytes (PEA)-15 is a novel regulator of adipose tissue expansion

Pola J. Verschoor[1], Fiona H. Greig[1], Justin J. Rochford[1,2], Giovanni Levate[1], Mirela Delibegovic[1], Dawn Thompson[1], Alasdair Leeson-Payne[2], Ruta Dekeryte[1], Ruth Banks[1], Joe W. Ramos[3], Graeme F. Nixon[1]

[1]Aberdeen Cardiovascular & Diabetes Centre, University of Aberdeen, Institute of Medical Sciences, Aberdeen, UK

[2] Rowett Institute, University of Aberdeen, Aberdeen, UK

[3]University of Hawai'i Cancer Center, University of Hawai'i at Manoa, Honolulu, USA

Keywords - Phosphoprotein enriched in astrocytes-15; Adipose tissue; Atherosclerosis; Extracellular signal–regulated kinases 1/2

Corresponding author:

Graeme F. Nixon
Aberdeen Cardiovascular & Diabetes Centre
University of Aberdeen
Institute of Medical Sciences
Foresterhill
Aberdeen
AB25 2ZD
United Kingdom
Tel: +44 1224 473405
Email: g.f.nixon@abdn.ac.uk

Supplementary figure 1:

A

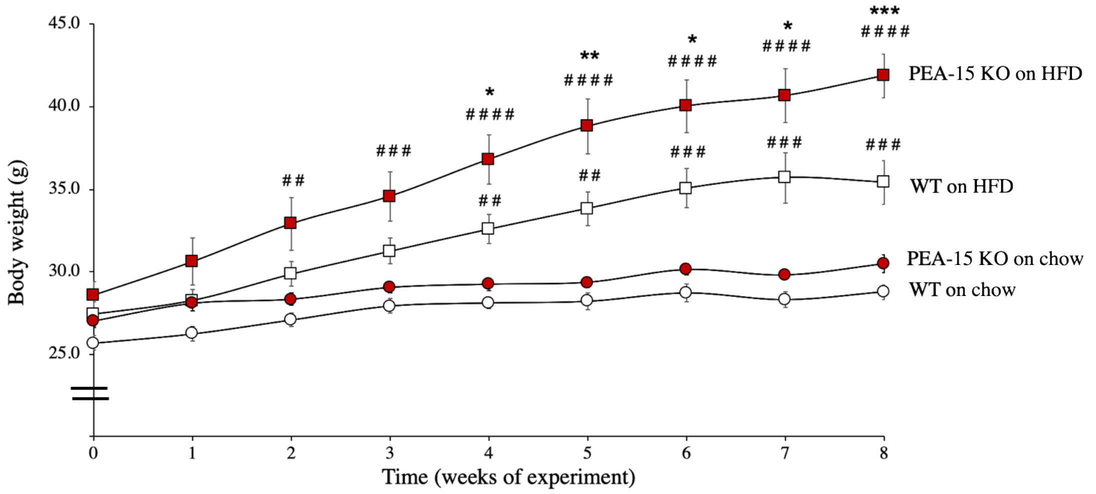

B

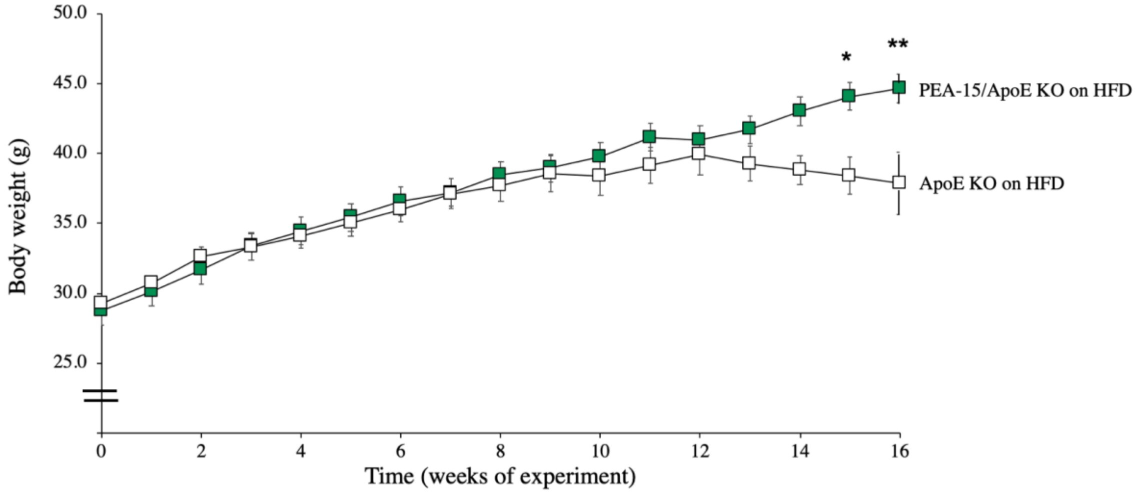

Supplementary figure 1: Body weight over the period of the diet. (A) Mean body weight of the PEA-15 KO and WT mice over the 8 weeks of chow or HFD. Graph displays body weight as mean±SEM, n=7. *P<0.05, **p<0.01 and ***p<0.001 when comparing genotypes and ##p<0.01, ###p<0.001 and ####p<0.0001 when comparing diets using two-way ANOVA and Bonferroni post hoc test. (B) Mean body weight of PEA-15/ApoE KO (n=11) and ApoE KO (n=7) mice over the 16 weeks of HFD. **P<0.01 and ****p<0.0001 using two-way ANOVA and Bonferroni post hoc test.

Supplementary figure 2:

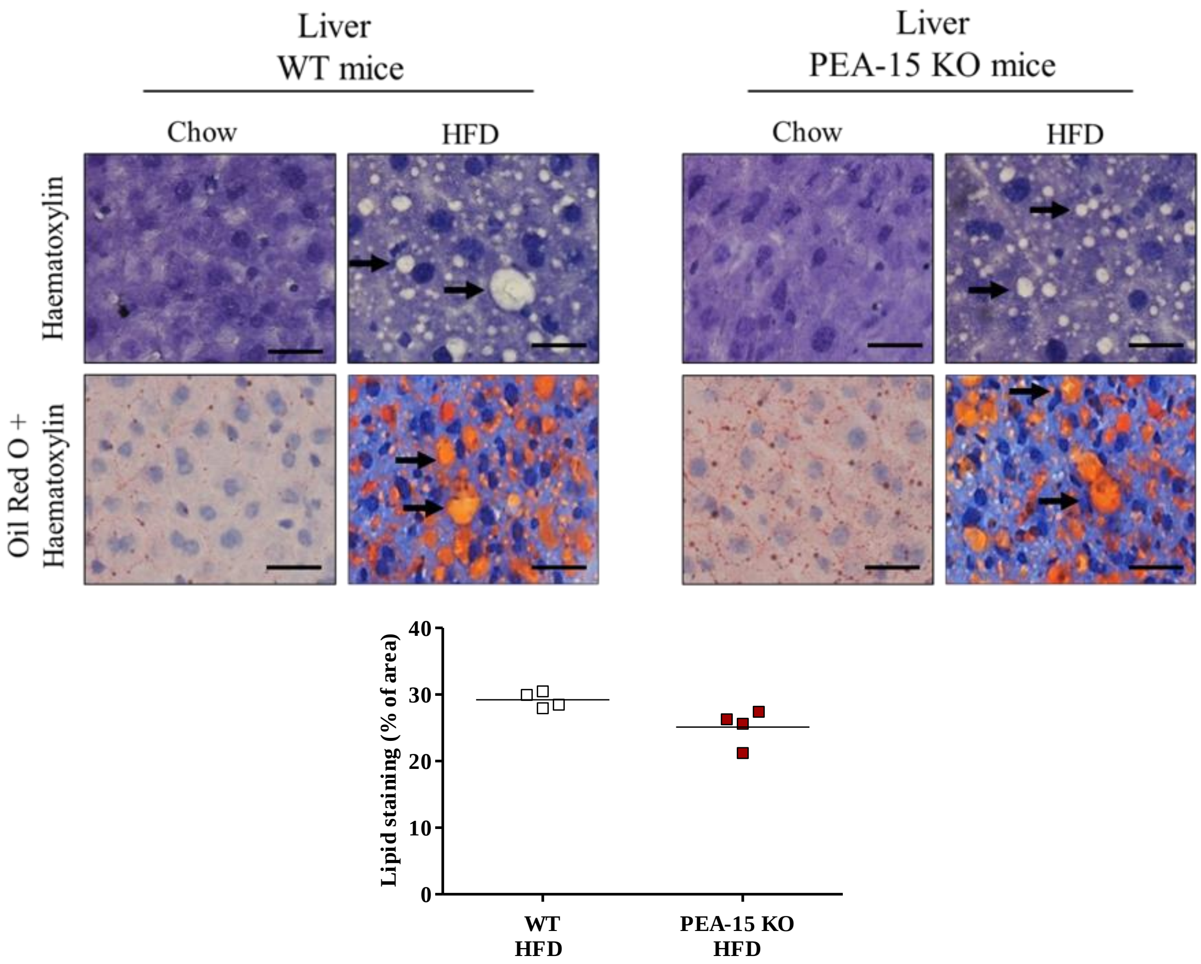

Supplementary figure 2: Representative bright field images of liver sections of WT and PEA-15 KO mice after 8 weeks of chow or HFD. Cryosections were stained with haematoxylin (blue) and Oil Red O (red). Arrows indicate examples of lipid vacuoles. Scale bar = 50 µm. Graph displays the quantification of lipid accumulation in livers of HFD-fed mice using the Oil-red O stained images of six representative pictures per animal, measured blinded to mouse genotype, n=4. All graphs were produced using GraphPad Prism version 9.0.0 for Windows, GraphPad Software, San Diego, California USA, www.graphpad.com.

Supplementary figure 3:

A

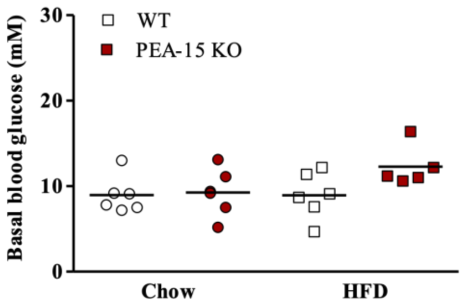

B

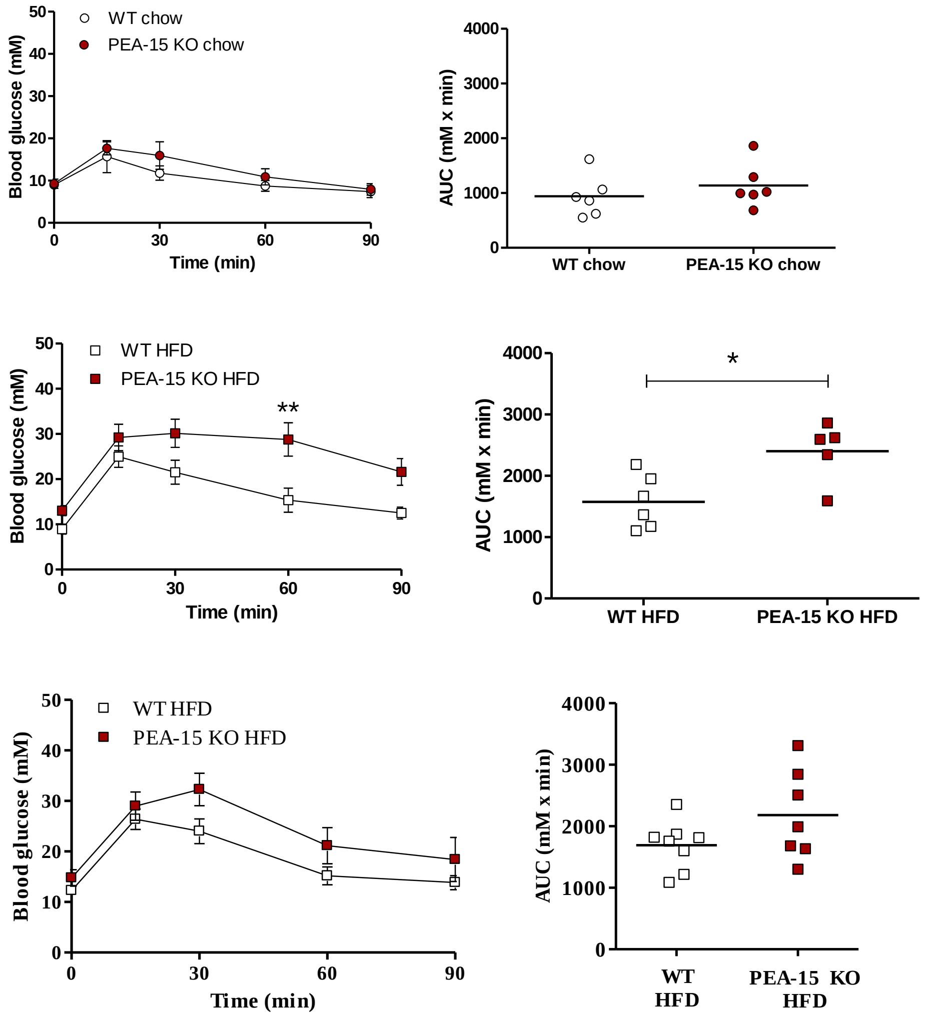

Supplementary figure 3: (A) Fasted (5h) basal glucose measurement of WT mice and PEA-15 KO mice after eight weeks of diet, n=5-6. (B) Fasted (5h) glucose measurements of WT mice and PEA-15 KO mice challenged with a glucose bolus, *p<0.05 and **p < 0.01 using two-way ANOVA and Bonferroni post hoc test, n=5-8. Top: after eight weeks of chow. Center: after eight weeks of HFD. Bottom: after 11 weeks of HFD. Graphs on the right are the corresponding area under the curve (AUC) calculations of blood glucose levels, Student t-test, n=5-8. All data are presented as the mean±SEM. All graphs were

produced using GraphPad Prism version 9.0.0 for Windows, GraphPad Software, San Diego, California USA, www.graphpad.com.

Supplementary figure 4:

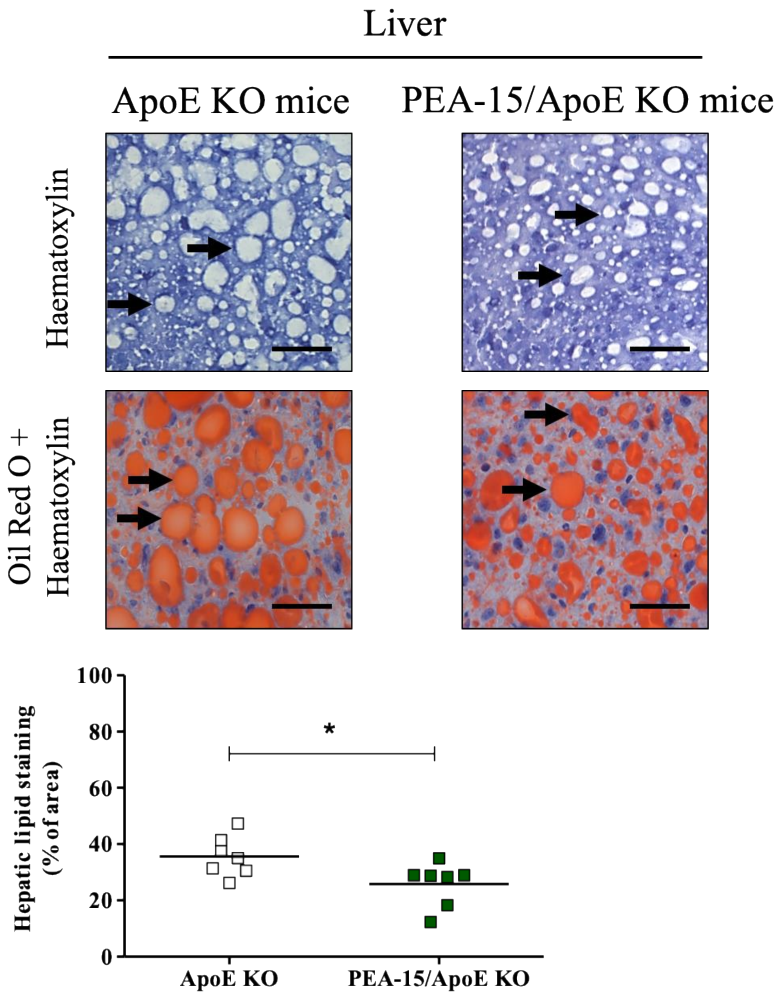

Supplementary figure 4: Representative bright field images of liver sections of ApoE KO and PEA-15/ApoE KO mice after 16 weeks of HFD. Cryosections were stained with haematoxylin (blue) and Oil Red O (red). Arrows indicate examples of lipid vacuoles. Scale bar = 50 µm. Graph displays the quantification of lipid accumulation in livers using the Oil-red O stained images of six representative pictures per animal, measured blinded to mouse genotype, n=7. *P<0.05 using Student t-test. All graphs were produced using GraphPad Prism version 9.0.0 for Windows, GraphPad Software, San Diego, California USA, www.graphpad.com.

Supplementary figure 5:

This figure shows the full original uncropped immunoblot images of Figure 1A (results).

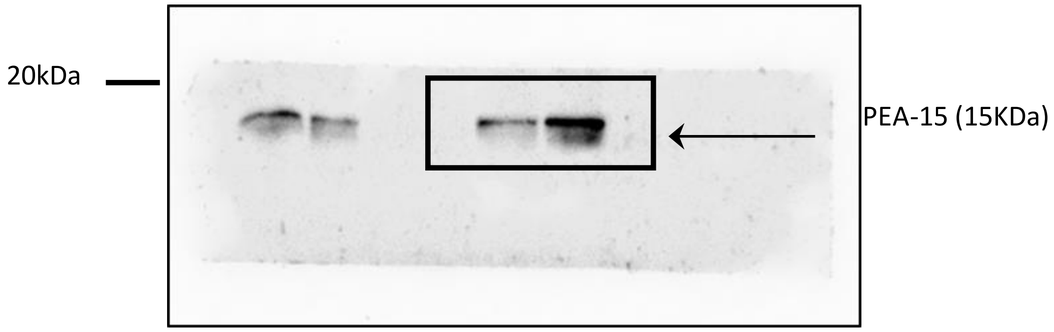

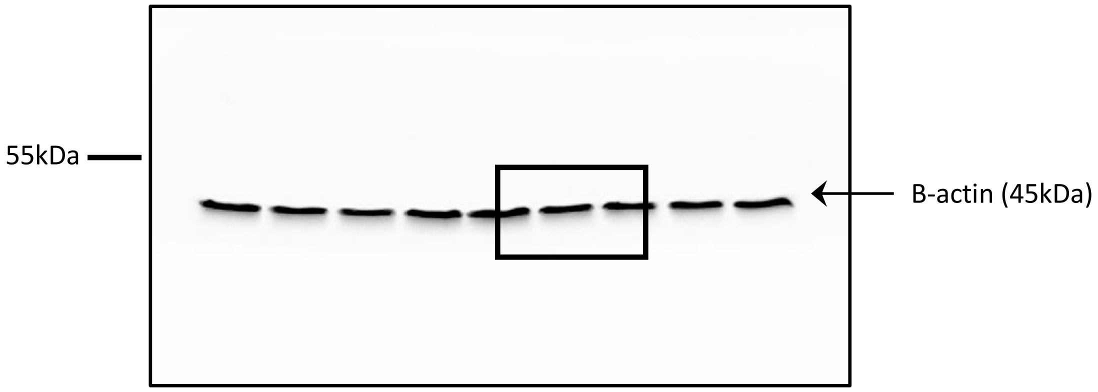

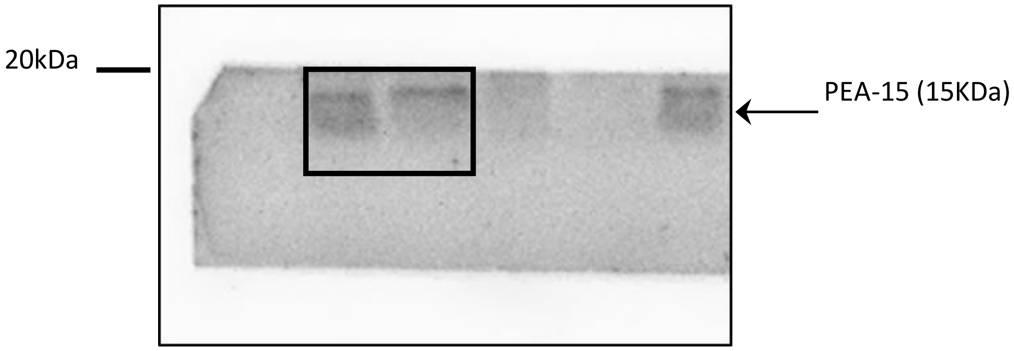

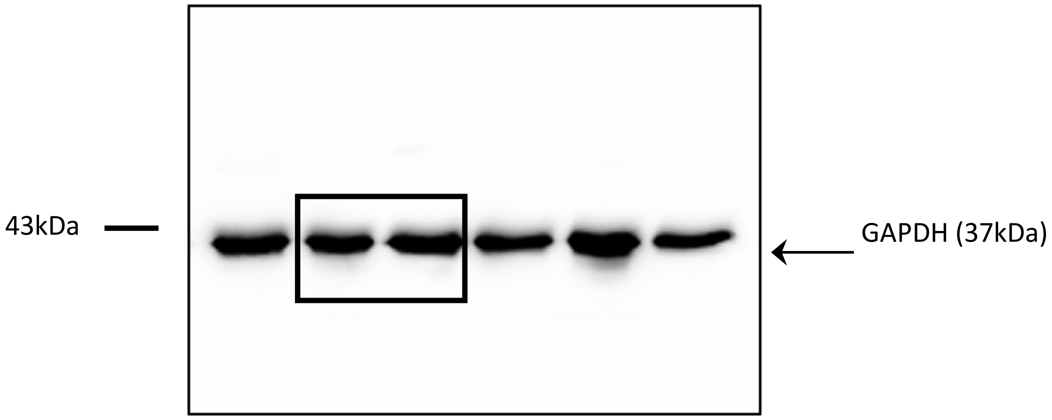

Supplementary figure 6:

This figure shows the full original uncropped immunoblot images of Figure 2B (results).

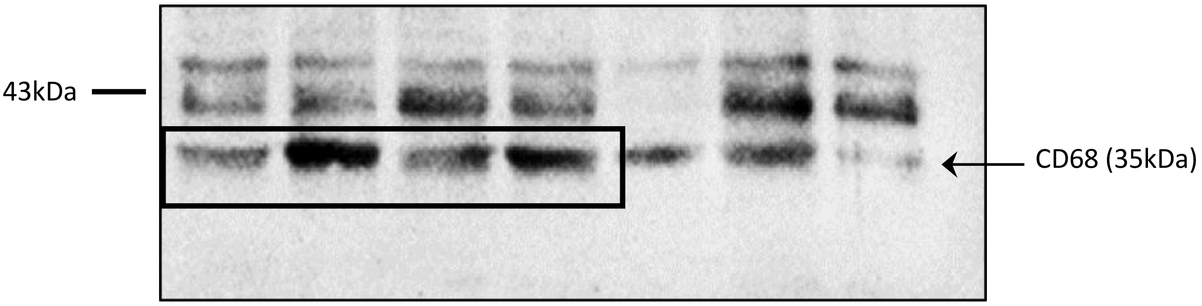

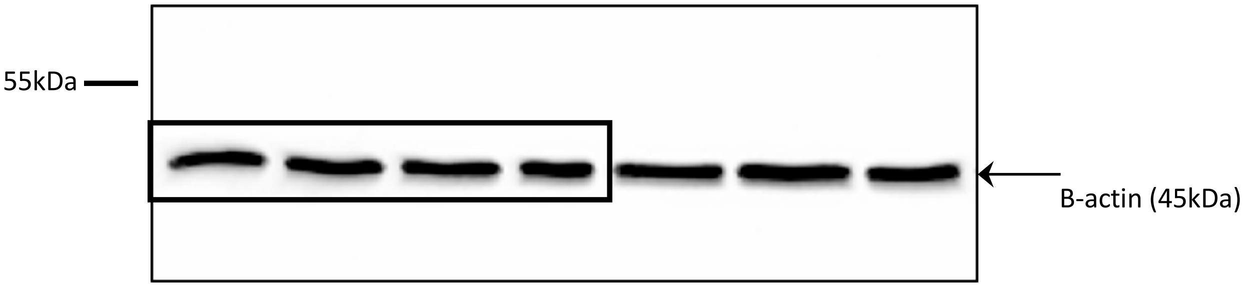

Supplementary figure 7:

This figure shows the full original uncropped immunoblot images of Figure 2C (results).

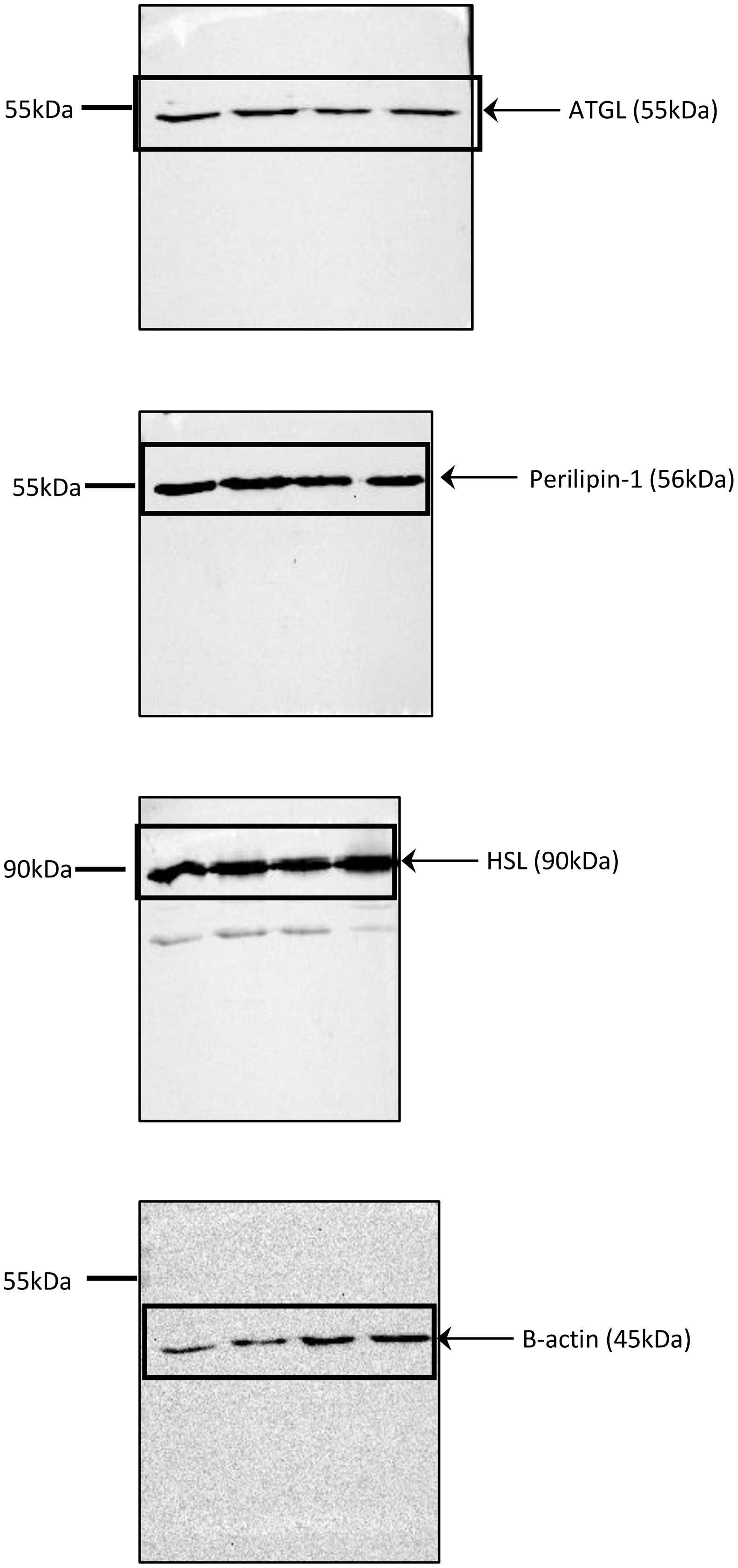

Supplementary figure 8:

This figure shows the full original uncropped immunoblot images of Figure 3B (results).

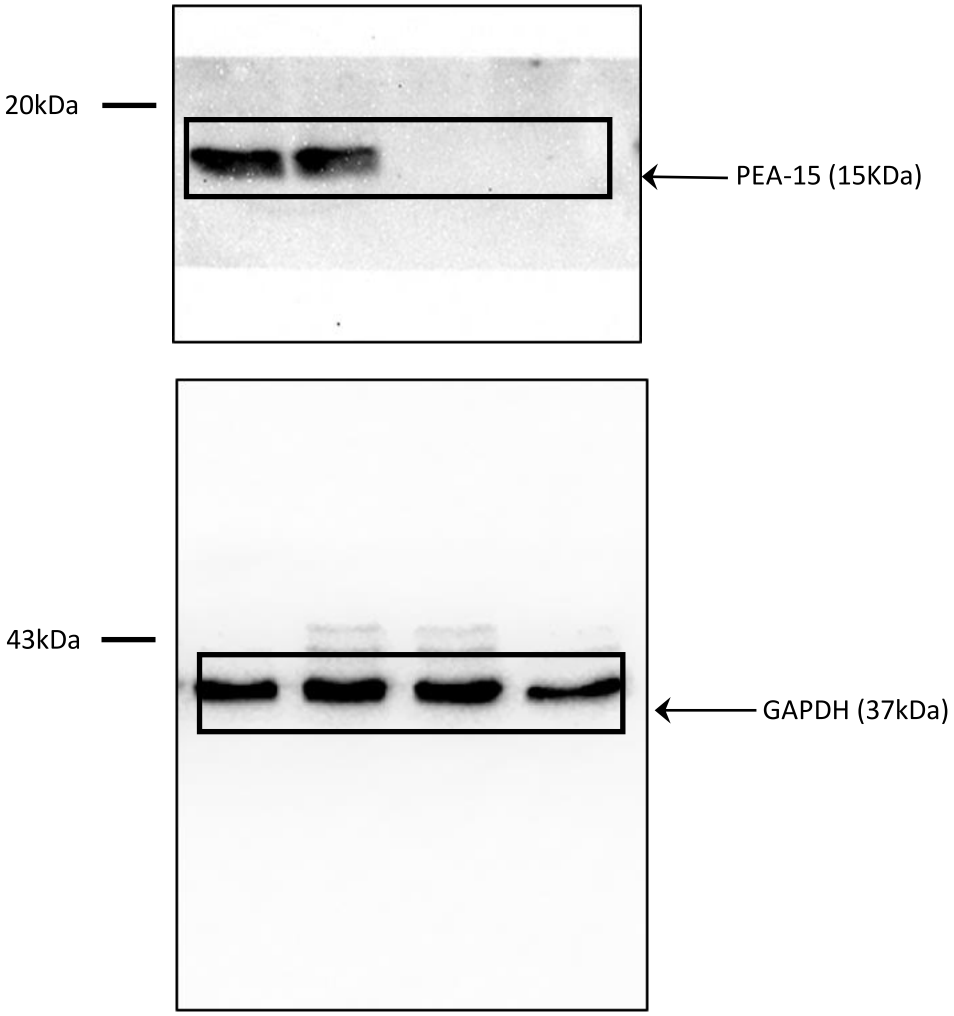

Supplementary figure 9:

This figure shows the full original uncropped immunoblot images of Figure 3G (results).

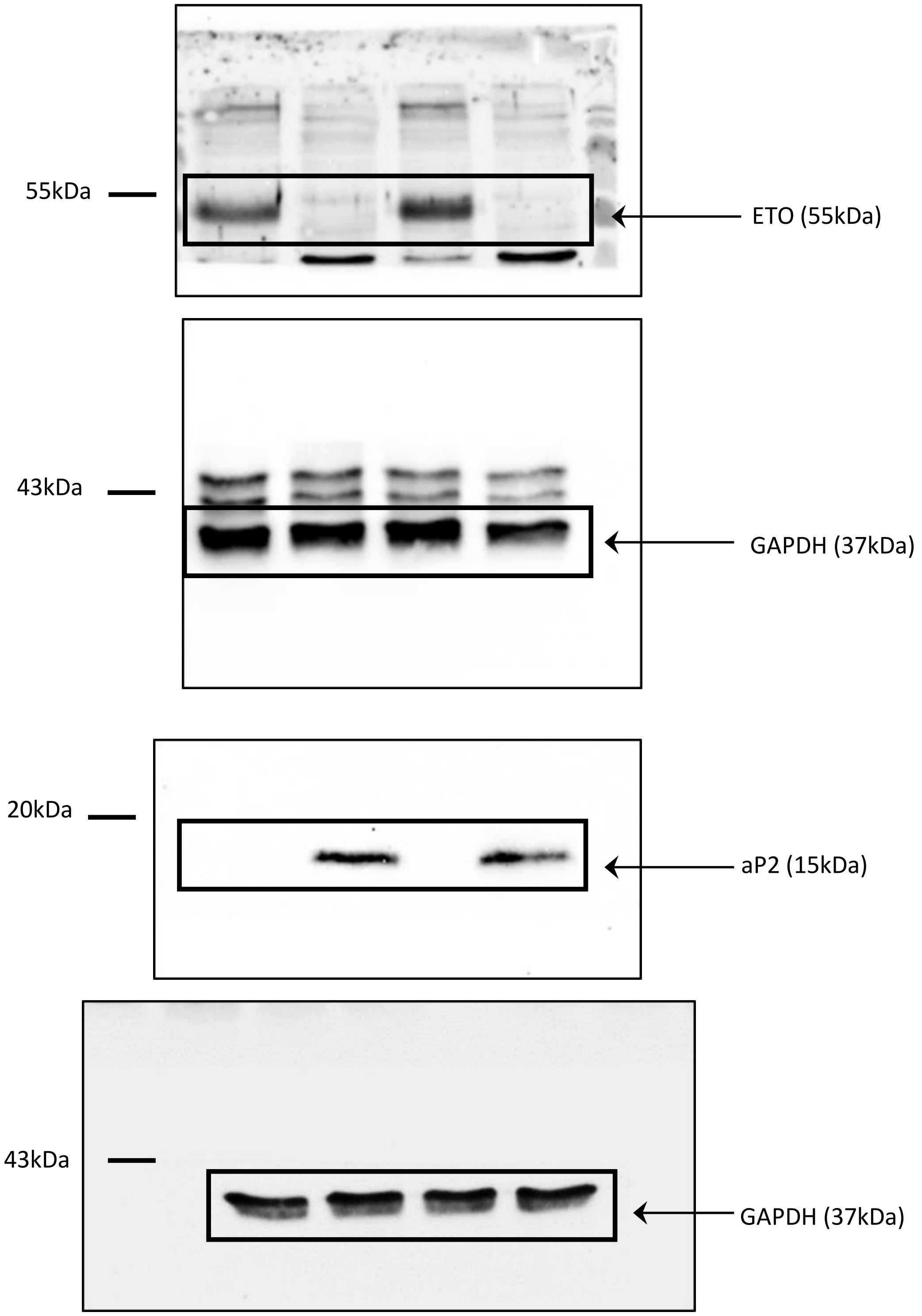

Supplementary figure 10:

This figure shows the full original uncropped immunoblot images of Figure 4A (results).

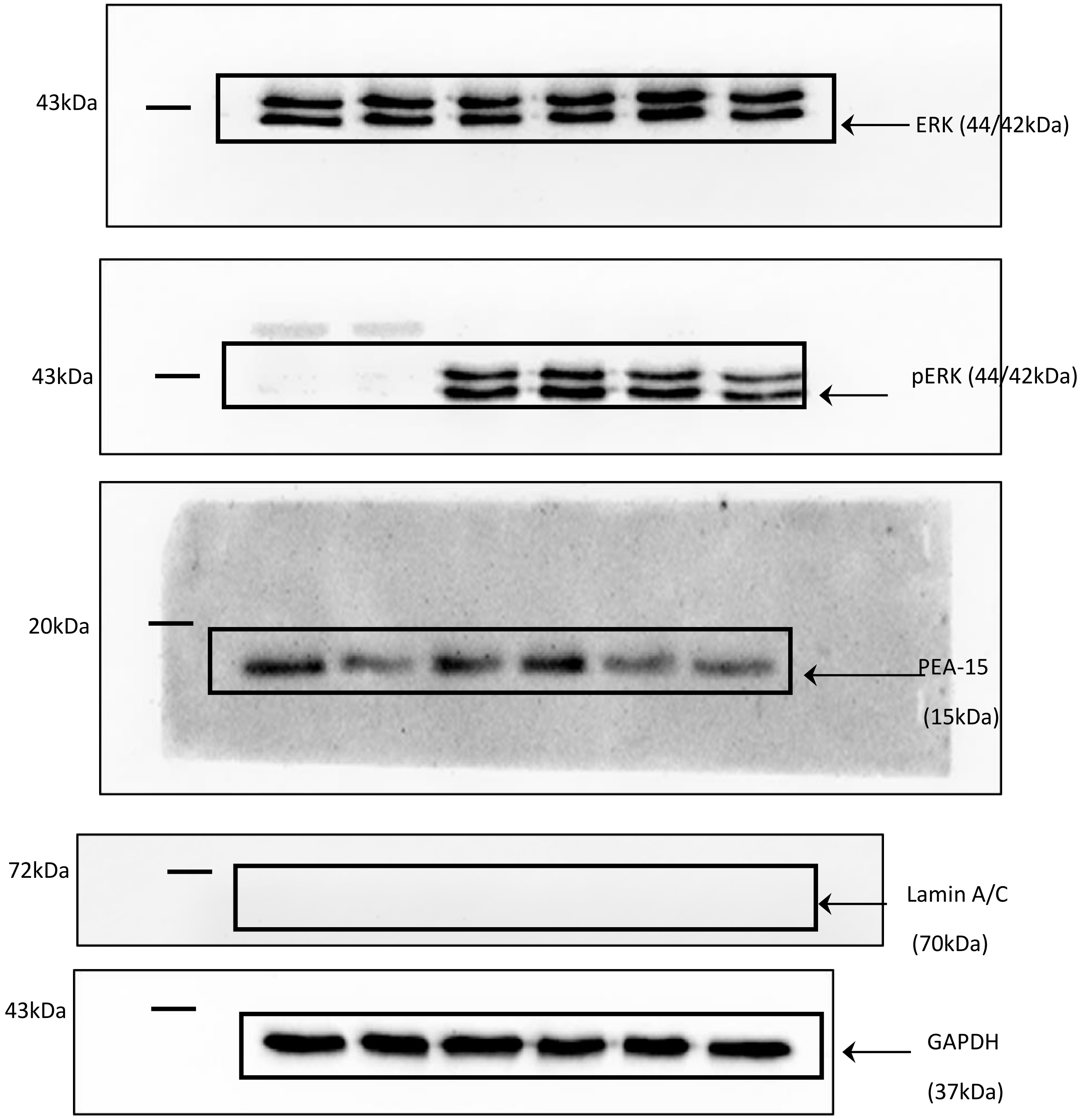

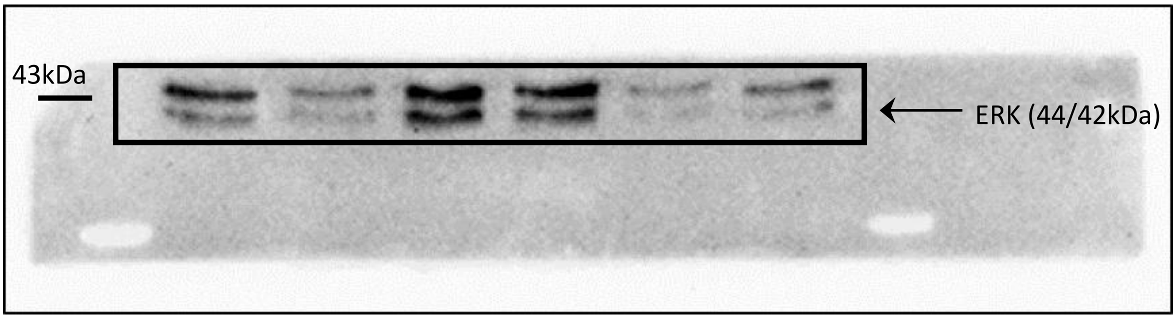
43kDa
ERK (44/42kDa)

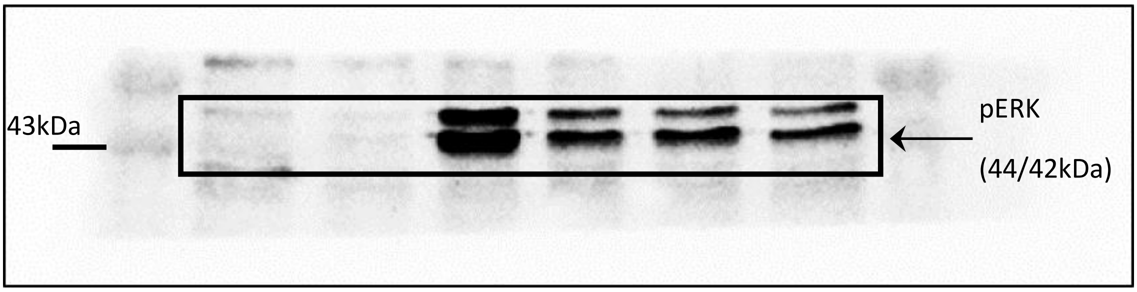
43kDa
pERK
(44/42kDa)

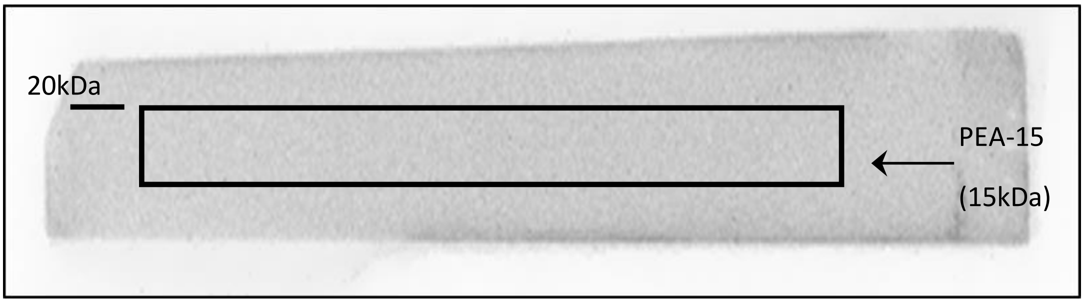
20kDa
PEA-15
(15kDa)

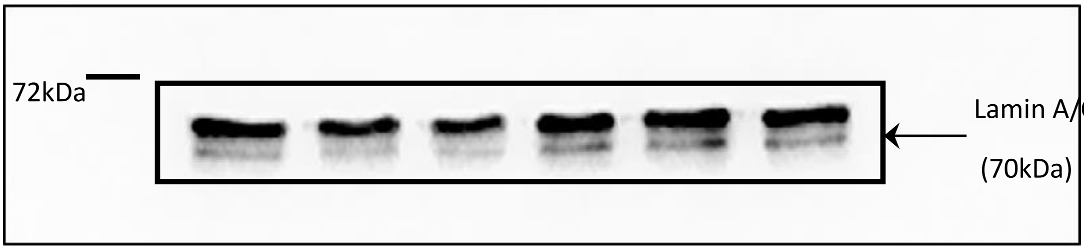
72kDa
Lamin A/C
(70kDa)

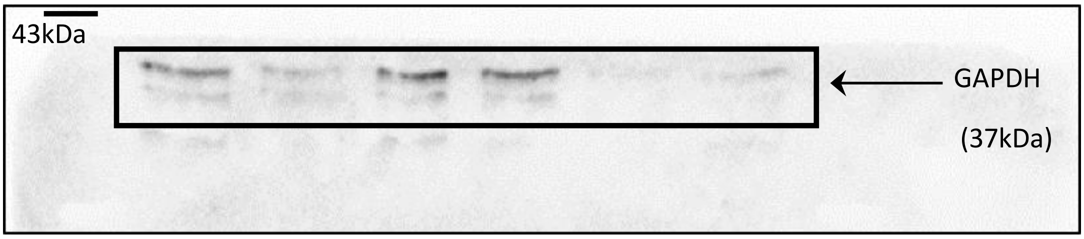
43kDa
GAPDH
(37kDa)

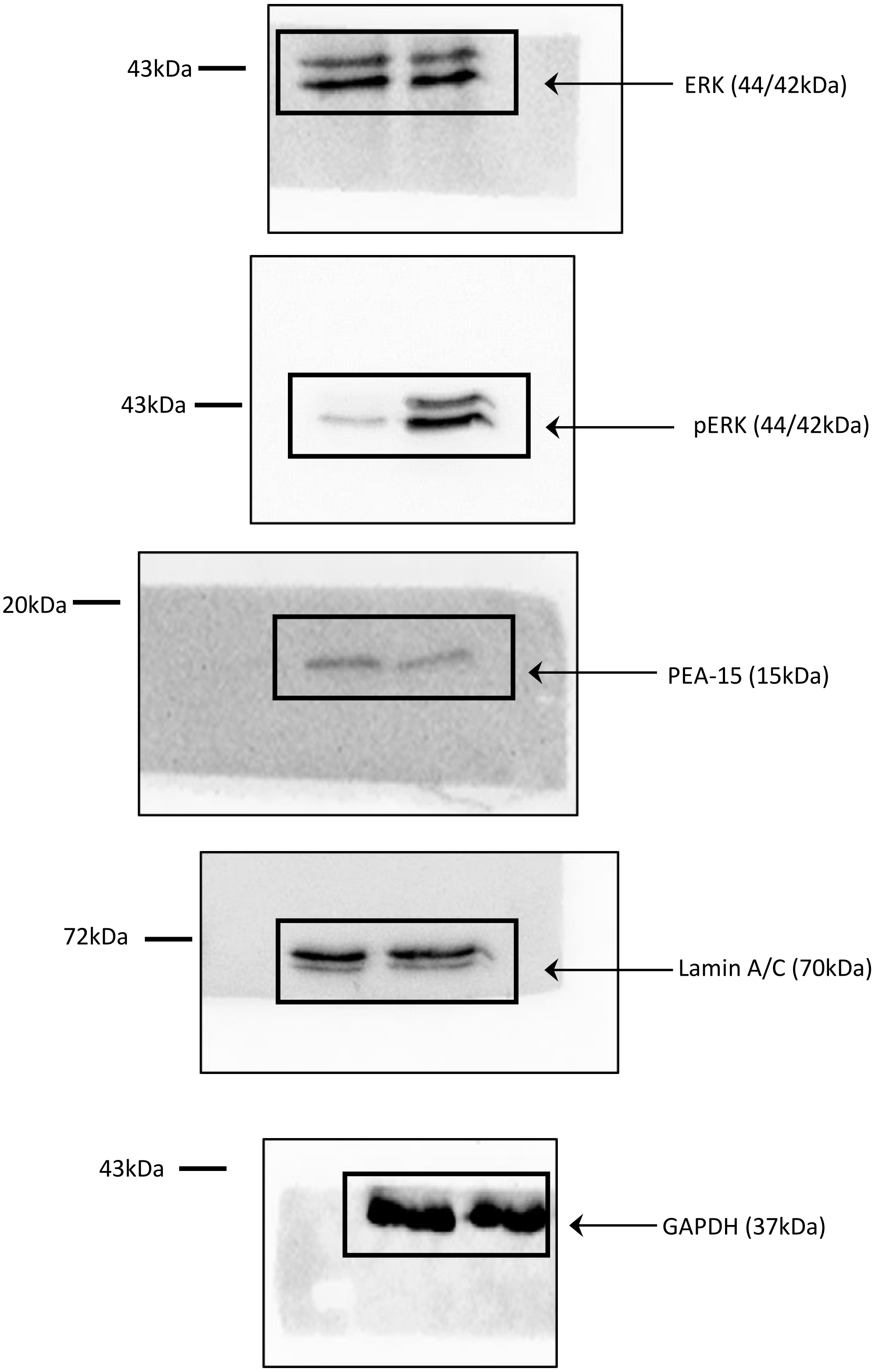
43kDa
ERK (44/42kDa)
43kDa
pERK (44/42kDa)
20kDa
PEA-15 (15kDa)
72kDa
Lamin A/C (70kDa)
43kDa
GAPDH (37kDa)

Supplementary figure 11:

This figure shows the full original uncropped immunoblot images of Figure 4B (results).

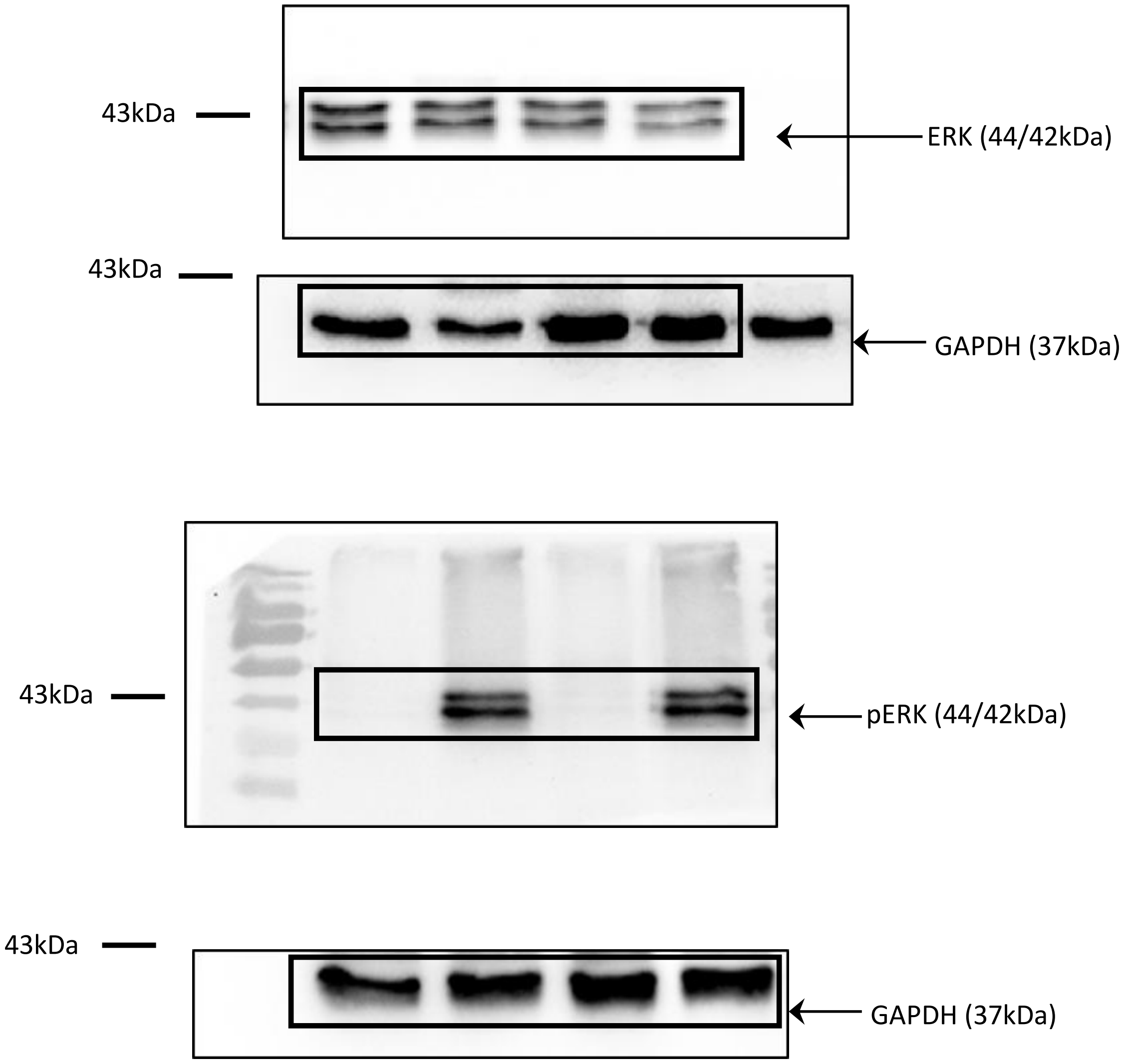

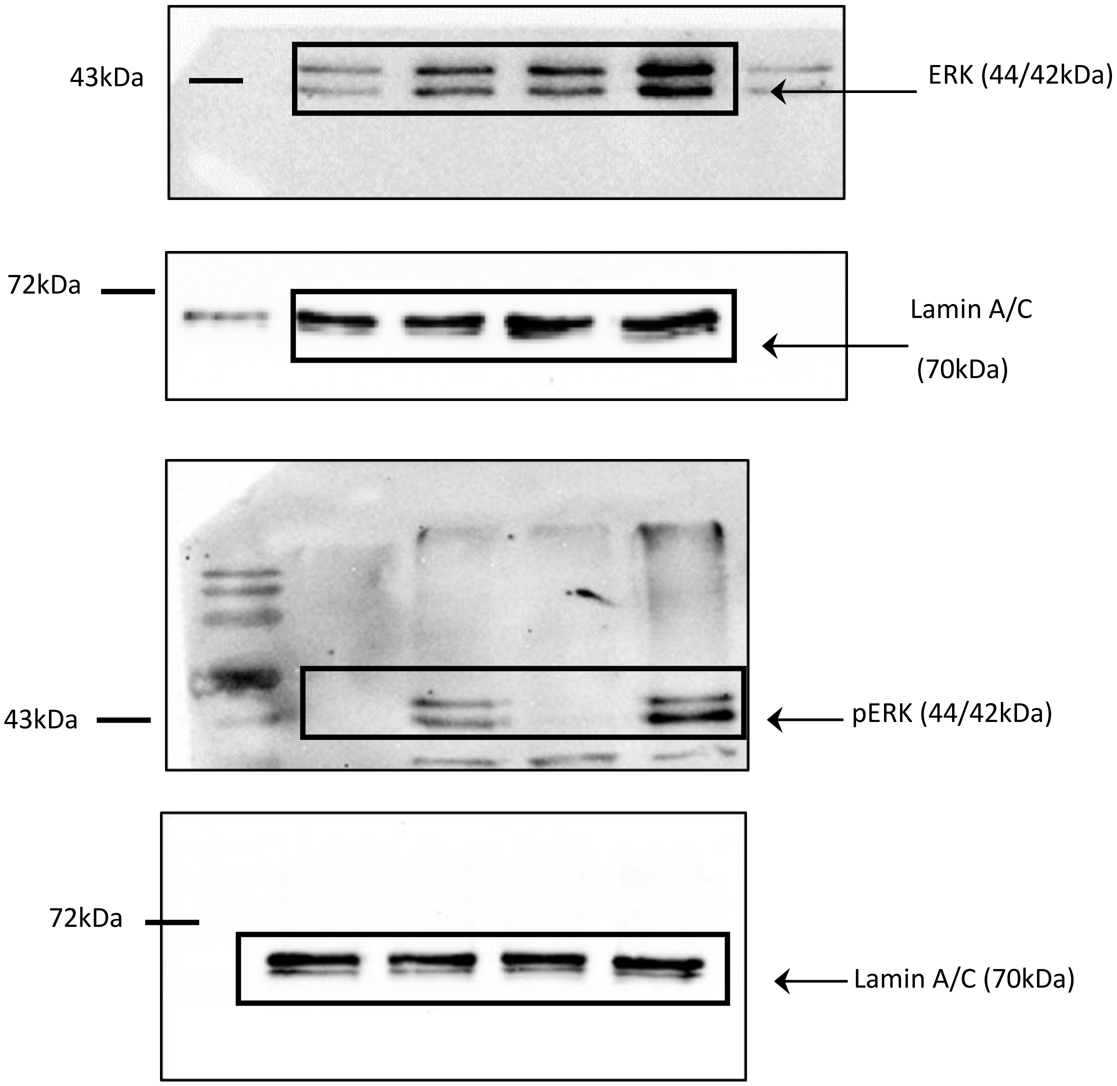
43kDa
ERK (44/42kDa)
72kDa
Lamin A/C
(70kDa)
43kDa
pERK (44/42kDa)
72kDa
Lamin A/C (70kDa)
